# On the Identity of Pear Psyllids in Iran: A Plea Against the Continued Misuse of *Cacopsylla pyricola* (Hemiptera: Psylloidea)

**DOI:** 10.3390/insects17090915

**Published:** 2026-09-01

**Authors:** Mohammadreza Lashkari, Liliya Štarhová Serbina

**Affiliations:** 1 Department of Biodiversity, Institute of Science and High Technology and Environmental Sciences, Graduate University of Advanced Technology, Kerman 7631818356, Iran; 2 Leibniz Institute for the Analysis of Biodiversity Change, Museum of Nature Hamburg, 20146 Hamburg, Germany; l.serbina@leibniz-lib.de; 3 Center for Integrative Biodiversity Discovery, Museum für Naturkunde, 10115 Berlin, Germany

**Keywords:** Psyllidae, *Pyrus*, jumping plant lice, pests, identification key, GenBank species misidentifications, molecular barcodes

## Abstract

Pear psyllids are among the most serious pests of cultivated pear. In the past, repeated misidentifications and the uncritical application of species names led to considerable confusion in biodiversity records and pest monitoring of pear psyllids. Although several revisions clarified many of these issues, misidentifications have persisted, particularly in records attributed to *Cacopsylla pyricola* (Foerster) in Iran, which may result in serious problems in quarantine and pest management. Currently, 34 species of *Cacopsylla* are known to be associated with *Pyrus* spp., five of which are confirmed to occur in Iran, while the occurrence of *C. pyricola* in the country remains likely but unconfirmed. In this study, we demonstrate that the examined *Cacopsylla* species differ significantly in their COI barcodes, which can be reliably used for species identification. The COI sequences for *C. permixta* are published here for the first time. Our results suggest that the GenBank and literature records of *C. pyricola* from Iran likely represent misidentifications of other species that occur throughout the pear-producing regions of Iran. These misidentifications likely resulted from the application of species names without adequate morphological or molecular confirmation. Our findings highlight the need for careful validation of species records and demonstrate that GenBank sequences should be used cautiously unless they are linked to reliably identified voucher specimens.

## Simple Summary

Pear psyllids are important pests of pear orchards, and some species are difficult to identify because they are very similar in appearance. Incorrect identification can affect pest management and quarantine decisions. In this study, we used DNA barcoding to identify pear psyllid species occurring in Iran. We also provide the first DNA barcode sequences for *Cacopsylla permixta*. Our results showed that the GenBank records of *C. pyricola* from Iran, which were examined in this study, were based on misidentified specimens belonging to other species. Our findings highlight the importance of accurate species records and careful use of GenBank databases. The occurrence of *C. pyricola* in Iran should be verified through targeted sampling and examination of reliably identified voucher specimens. Further studies are also needed to determine whether pear psyllid species can act as vectors of phytoplasma in Iran.

## 1. Introduction

*Pyrus* (Rosaceae), a genus of trees and shrubs with 73 valid species in the Palaearctic and Oriental regions [1], represents an economically significant crop in the temperate and subtropical regions of the world. Pear species, mostly *Pyrus communis* subsp. *communis* in Europe and North America, and *P. bretschneideri*, *P. pyrifolia* and *P. ussuriensis* in China rank among the five most widely produced fruits in the world [2]. In 2022, the estimated global pear production was over 26 million metric tonnes, with approximately 73% originating from China [3]. Pear is an important fruit crop in Iran, where orchards covered approximately 3273 hectares and produced about 95.4 thousand tonnes in 2024, ranking the country 21st among approximately 80–90 pear-producing nations worldwide [3]. Commercial cultivation occurs in the provinces of Khorasan, Tehran, Esfahan, West and East Azerbaijan, Zanjan, Qazvin, Kurdistan, Lorestan and Semnan [4].

Pear psyllids (Psyllidae: Psyllinae: *Cacopsylla*) are among the most serious pests of pear. They weaken plants through excessive removal of phloem sap and impede the development of flowers by feeding on flower buds; they also indirectly harm plants by secreting honeydew, which promotes the growth of sooty mould and reduces photosynthesis [5,6]. Moreover, several species act as vectors of ‘*Candidatus* Phytoplasma pyri’, the causal agent of Pear Decline [7,8]. Pear Decline has also been detected in Iran [9,10], although no psyllid species has yet been confirmed as a vector of the phytoplasma in the country, unlike in Europe and the USA [7,8,11].

Currently, 34 species of *Cacopsylla* are known to live on *Pyrus* spp. in the Palaearctic region, five of which are confirmed to occur in Iran, while the presence of *C. pyricola* still requires verification. Several species are polyvoltine, e.g., the members of the *Cacopsylla pyri*-group, and produce morphologically distinct summer and winter generations [12]. In contrast, members of the *C. pyrisuga*-group are univoltine and migrate to conifers to overwinter. Seasonal dimorphism within species and morphological similarity among species have historically led to taxonomic confusion, which was largely resolved by Burckhardt and Hodkinson (1986) [12] for Western Palaearctic taxa and by Yang et al. (2004) [13], Luo et al. (2012) [14] and Cho et al. (2017) [15] for Eastern Palaearctic taxa. Despite these revisions, confusion persists in published biodiversity and pest-monitoring data. Misidentifications in genetic databases, particularly GenBank, remain a major source of error.

The presence of five pear psyllid species in Iran has been well documented [12]: *Cacopsylla bidens* (Šulc, 1907), *C. notata* (Flor, 1861), *C. permixta* Burckhardt and Hodkinson, 1986, *C. pyri* (Linnaeus, 1758) and *C. pyrisuga* (Foerster, 1848). Another species, *C. pyricola* (Foerster, 1848), has been repeatedly reported from Iran, between 1961 and 2025, often based on questionable records or apparent misidentifications. The pear psyllids of Iran belong to two species groups: the *Cacopsylla pyri*-group (polyvoltine, overwintering as adults on the host plant or in leaf litter around the host) and the *C. pyrisuga*-group (univoltine, overwintering as adults on conifers). The *Cacopsylla pyri*-group is also characterised by a distinct morphological dimorphism between winter and summer generations. The summer form of *C. pyri* is distinguished from the winter form by the absence of brown clouding in the forewing, the presence of denser and more widespread forewing spinules, and its smaller size [12,16]. In *C. bidens* and *C. permixta*, the dimorphism affects mostly the body colour and size, with the winter form darker and larger than the summer form, which is lighter, variegated and smaller, while in *C. notata*, the seasonal dimorphism is very slight, affecting mostly the size [12].

The present study addresses these issues by analysing GenBank records, published literature and newly generated sequences, and by providing an illustrated morphological identification key to the Iranian pear psyllids, with the aim of helping pear growers and researchers accurately identify these species and avoid future misidentifications.

## 2. Materials and Methods

### 2.1. Literature Review

A structured literature search was conducted to compile previously published records of *C. pyricola* from Iran. Searches were performed across major Iranian and international scientific databases. The retrieved records were classified into two periods, before and after the taxonomic revision of the West Palaearctic pear psyllids by Burckhardt and Hodkinson (1986) [12], which provided a revised taxonomic framework for *Pyrus*-feeding *Cacopsylla* species. All relevant information was subsequently compiled in a standardised Excel database and categorised into four source types: (i) primary references, (ii) secondary references, (iii) GenBank records, and (iv) relevant online sources. For each record, available bibliographic, geographic, host plant, species identification, depository, and molecular information, including GenBank accession numbers, was extracted.

### 2.2. Material Examined

Material examined in this study came from two main sources: field collections and entomological collections. Field work was conducted by ML between 2022 and 2024 in pear orchards in Iran. Specimens were collected from plants using a sweep net and an aspirator. Collected specimens were stored in 96% ethanol at −20 °C for DNA extraction. For the molecular study, two specimens of *Cacopsylla permixta* (1 male, 1 female), identified by Daniel Burckhardt, were examined. Both specimens were collected from *Pyrus* sp. in Iran (Kerman Province, Mahan), on 5 September 2023 by M. Lashkari and are deposited in NHMB.

Additional material examined was preserved in the following collections: KGUT—Department of Biodiversity, Institute of Science and High Technology and Environmental Sciences, Graduate University of Advanced Technology, Kerman, Iran; NHMB—Naturhistorisches Museum, Basel, Switzerland; and MHNG—Muséum d’histoire naturelle, Geneva, Switzerland.

The morphological terminology follows Bastin et al. (2023) [17].

### 2.3. DNA Extraction, PCR and Sequencing

DNA from *Cacopsylla permixta* was extracted non-destructively from whole specimens using a DNeasy Blood & Tissue Kit (QIAGEN, Hilden, Germany). A fragment of the *cytochrome oxidase c subunit I* (COI) gene was amplified for DNA barcoding of *C. permixta* using the primer set described by Folmer et al. (1994) [18] and Bastin et al. (2023b) [19], LCOP-F (5′-AGAACWAAYCATAAAAYWATTGG-3′) and HCO2198 (5′-TAAACTTCAGGGTGACCAAAAAATCA-3′), producing an expected amplicon of 658 bp. The primers were modified to include 5′ tag sequences described by Srivathsan et al. (2024) [20].

PCR conditions were as follows: 95 °C for 3 min; 40 cycles of 95 °C for 1 min, 48 °C for 30 s and 72 °C for 1 min; followed by 72 °C for 5 min. The resulting amplicons were sequenced using an Oxford Nanopore Technologies (ONT) MinION R10.4.1 flow cell on a GridION, using the SQK-LSK114 Ligation Kit v14 (Oxford Nanopore Technologies, Oxford, UK). Sequences were processed using ONTbarcoder 2.0, following the procedures developed by Srivathsan et al. (2021; 2024) [20,21].

In total, COI sequences from ten *Cacopsylla* species were included in the phylogenetic analysis. A total of 110 sequences were downloaded from GenBank. All sequences were aligned using ClustalW implemented in MEGA v11 [22]. Following multiple sequence alignment, a common overlapping region of 374 bp was identified across all sequences. Although this region was shorter than the standard length of the target DNA barcode, shorter sequences were retained rather than excluded to maximise the number of usable reference sequences and the representation of available taxa in the reference dataset. The 374 bp overlapping region was subsequently used for all analyses. The tree was constructed using the Neighbour-joining (NJ) algorithm with 1000 bootstrap replicates and the Kimura 2-Parameter (K2P) model.

Photos of dry-mounted specimens were taken by Dr. Daniel Burckhardt with a Leica Digital Camera DFC320 mounted on a Leica MZ12 dissecting microscope (Leica Microsystems (Switzerland) Ltd., Heerbrugg, Switzerland). CorelDRAW Graphics Suite v21 (Corel Corporation, Ottawa, ON, Canada) was used to redraw the morphological structures illustrated in Burckhardt and Hodkinson (1986) [12] for improved clarity and publication-quality presentation.

## 3. Results

### 3.1. Records of Cacopsylla pyricola in Iran

The literature review revealed numerous records of *Cacopsylla pyricola* from Iran, dating from 1961 to 2025. These records originated from a wide range of localities and were identified in primary and secondary references, GenBank records, and relevant web sources. A detailed summary of the records, their geographic origins, and the available taxonomic information is presented in Table 1.

### 3.2. Morphology of Iranian Pear Psyllids and Identification Keys

#### 3.2.1. Pear Psyllid Species Recorded from Iran

**Figure 1:**
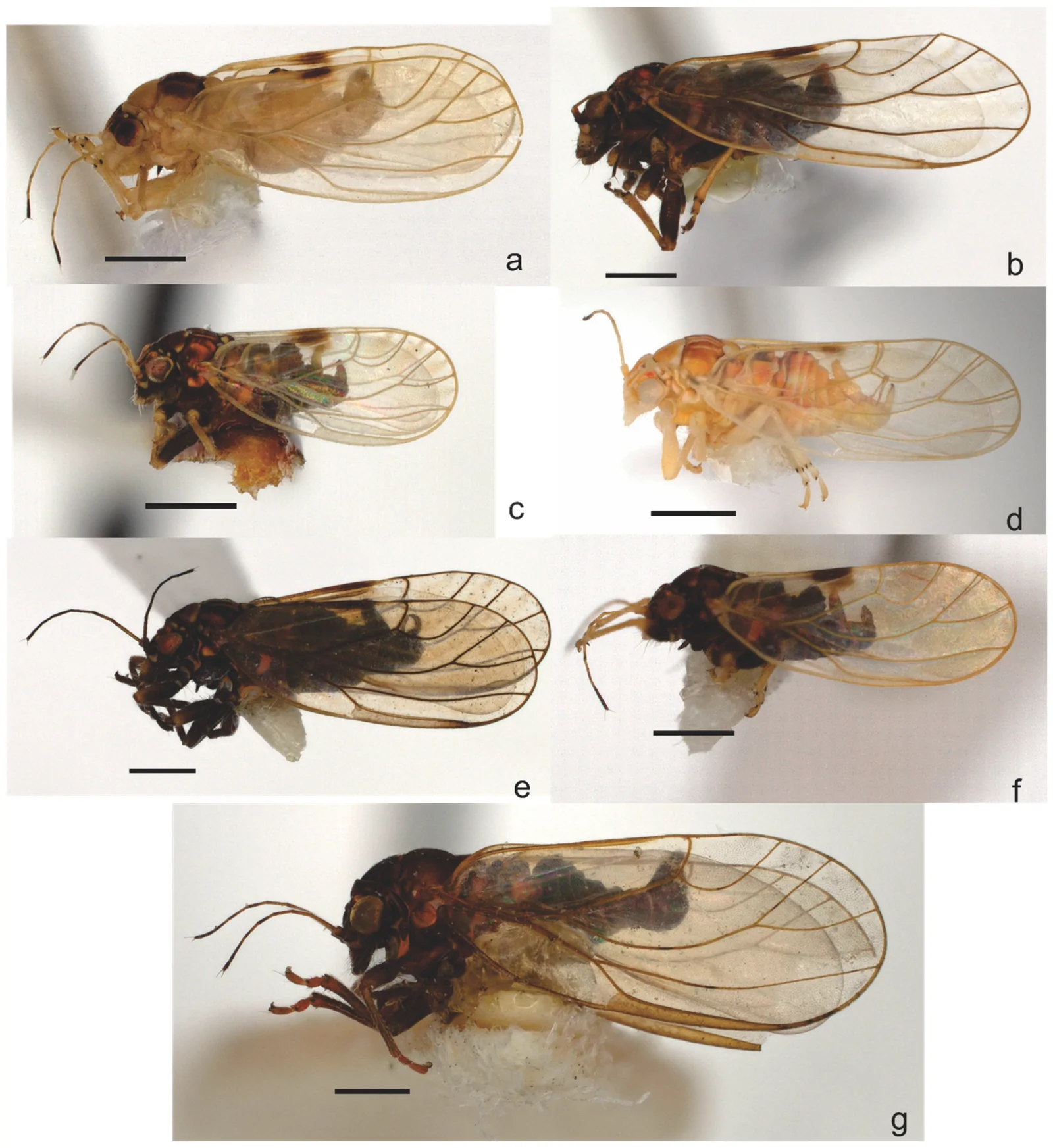
Adults of *Cacopsylla* spp., lateral view. (**a**) *Cacopsylla bidens* (summer form); (**b**) *C. bidens* (winter form); (**c**) *C. notata*; (**d**) *C. permixta*; (**e**) *C. pyri*; (**f**) *C. pyricola*; (**g**) *C. pyrisuga*. Scale bars = 0.5 mm.

**Figure 2:**
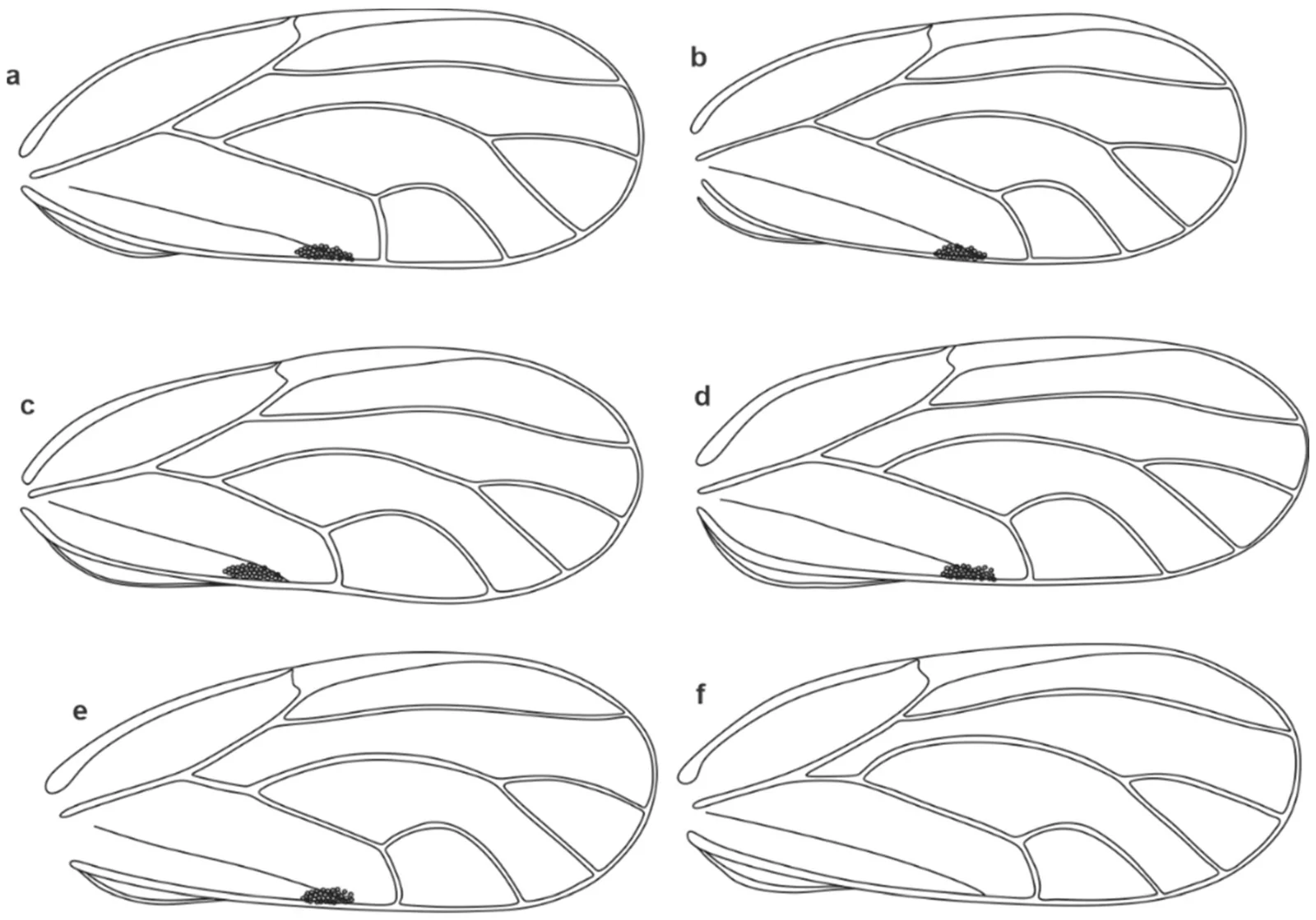
Forewings of *Cacopsylla* spp. (**a**) *Cacopsylla bidens*; (**b**) *C. notata*; (**c**) *C. permixta*; (**d**) *C. pyri*; (**e**) *C. pyricola*; (**f**) *C. pyrisuga*. Redrawn from Burckhardt and Hodkinson (1986) [12].

**Figure 3:**
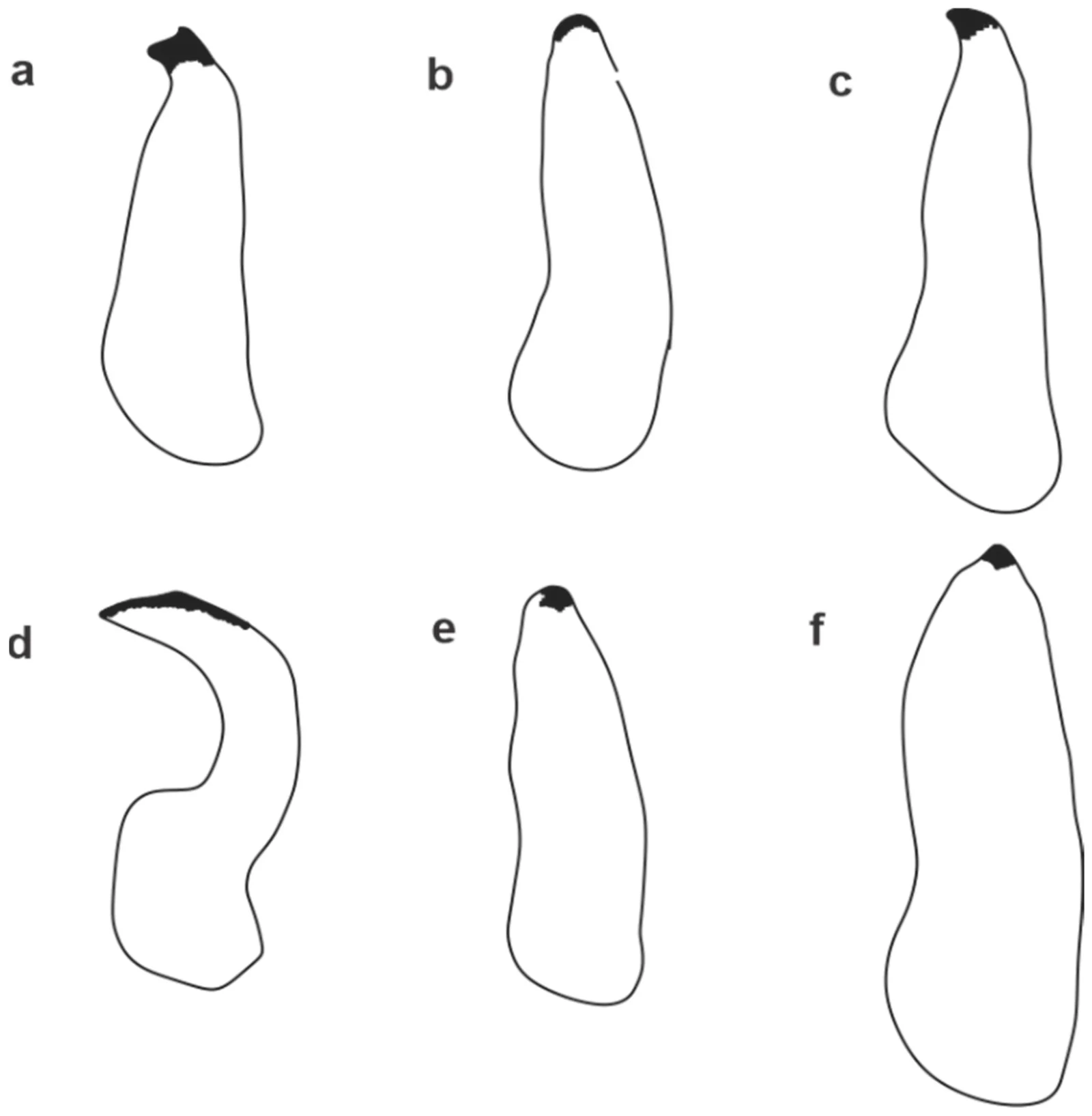
Inner surface of paramere of *Cacopsylla* spp., lateral view. (**a**) *Cacopsylla bidens*; (**b**) *C. notata*; (**c**) *C. permixta*; (**d**) *C. pyri*; (**e**) *C. pyricola*; (**f**) *C. pyrisuga*. Redrawn from Burckhardt and Hodkinson (1986) [12].

**Figure 4:**
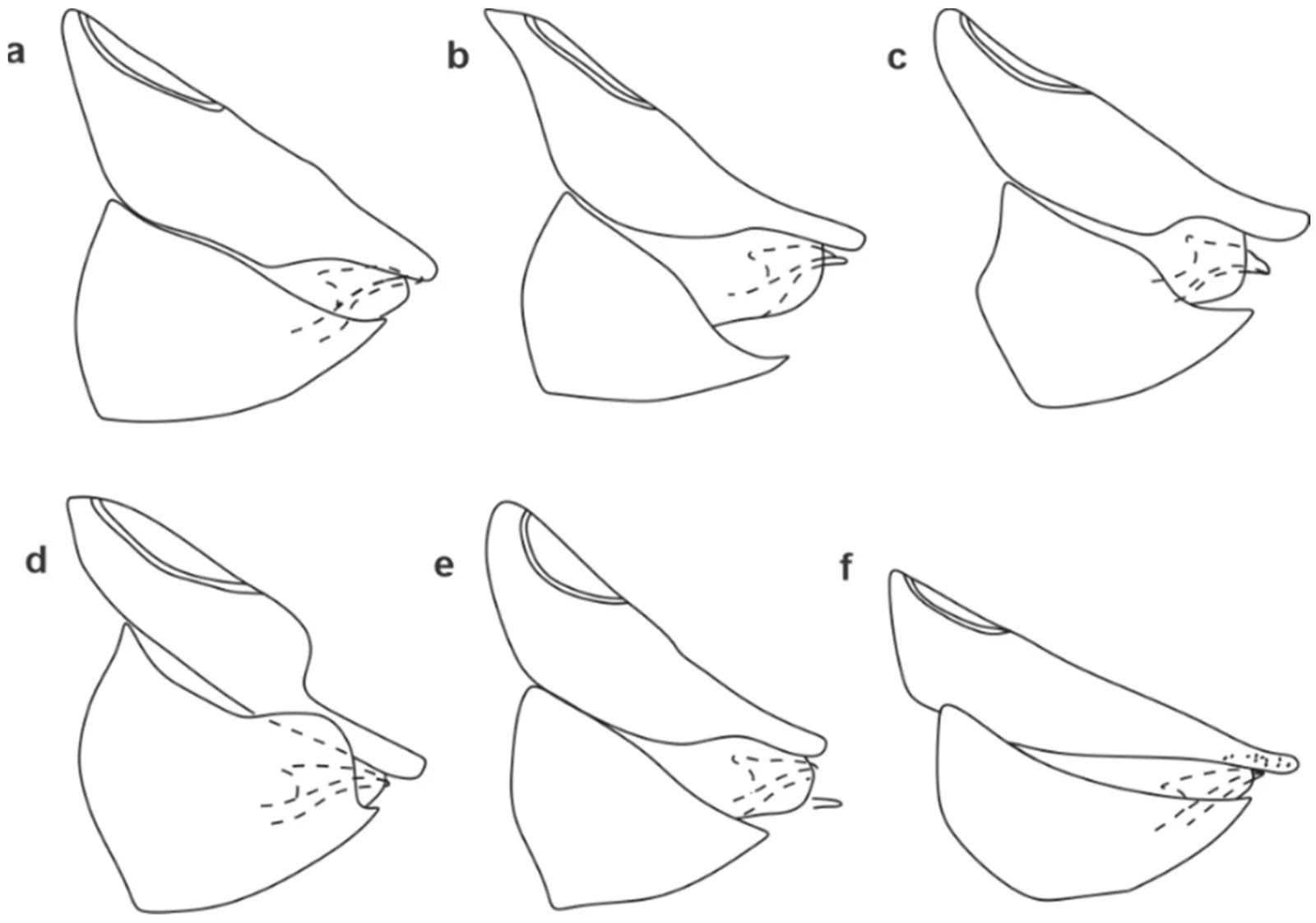
Female terminalia of *Cacopsylla* spp., lateral view. (**a**) *Cacopsylla bidens*; (**b**) *C. notata*; (**c**) *C. permixta*; (**d**) *C. pyri*; (**e**) *C. pyricola*; (**f**) *C. pyrisuga*. Redrawn from Burckhardt and Hodkinson (1986) [12].

***Cacopsylla bidens* (Šulc)**
  (Figure 1a,b, Figure 2a, Figure 3a and Figure 4a)

Material examined. IRAN, Alborz: 5 ♂, 5 ♀, Gachsar (36.03° N, 51.31° E), 2045 m, 16.ix.2022, leg. M Lashkari; 5 ♂, 5 ♀, Kondor (35.84° N, 51.10° E), 1874 m, 16.ix.2022, leg. M Lashkari; Esfahan: 5 ♂, 5 ♀, Abyaneh (33.58° N, 51.65° E), 1938 m, 19.x.2023, leg. M Lashkari; Razavi Khorasan: 5 ♂, 5 ♀, Mashhad (36.61° N, 59.69° E), 1286 m, 30.viii.2023, leg. M Lashkari.

Distribution in Iran. East Azerbaijan [33], Elborz (Karaj) [28], Esfehan [63], Kerman [64,65], Kurdistan [42,63], Razavi Khorasan [39], Tehran [28], West Azerbaijan [28,63], Zanjan [63], without details [12,15,66,67,68].

***Cacopsylla notata* (Flor, 1861)**
  (Figure 1c, Figure 2b, Figure 3b and Figure 4b)

Distribution in Iran. Kohgiluyeh and Boyer-Ahmad (Yasuj) [28], without details [15,69].

***Cacopsylla permixta* Burckhardt & Hodkinson, 1986**
  (Figure 1d, Figure 2c, Figure 3c and Figure 4c)

Material examined. IRAN, Kerman: Mahan (30.09° N, 57.21° E), 1908 m, 5.ix.2023, leg. M Lashkari; 5 ♂, 5 ♀, Bardsir (29.53° N, 56.66° E), 2595 m, 5.x.2022, leg. M Lashkari.

Distribution in Iran. Alborz [63], Golestan [28], North Khorasan [63], Tehran [63], Semnan [28].

***Cacopsylla pyri* (Linnaeus, 1758)**
  (Figure 1e, Figure 2d, Figure 3d and Figure 4d)

Distribution in Iran. East Azerbaijan [33], Tehran [70], Zanjan [28], without details [15,68].

***Cacopsylla pyrisuga* (Foerster, 1848)**
  (Figure 1g, Figure 2f, Figure 3f and Figure 4f)

Distribution in Iran. Elborz [28], Mazandaran (as *Psylla pyrisuga*) [71], without details [15].

#### 3.2.2. Misidentification

***C. pyricola* (Foerster, 1848)**
  (Figure 1f, Figure 2e, Figure 3e and Figure 4e)

Previous records of *C. pyricola* (Foerster, 1848) from Iran are considered doubtful and are summarised in Table 1.

#### 3.2.3. Key to Adults of Iranian Pear Psyllids; Modified from Burckhardt and Hodkinson (1986)

1. Forewings longer than 2.8 mm; apex of clavus not brown (Figure 2f). Paramere longer than distal segment of aedeagus (Figure 3f)································································································································································································***C. pyrisuga* (Foerster)**
  - Forewings shorter than 2.7 mm; if longer (in some *C. pyri* adults) then clavus with apical brown patch (Figure 2a–e). Paramere as long as or shorter than distal segment of aedeagus (Figure 3a–e)································································································································**2**
2. Paramere in profile sickle-shaped (Figure 3d). Dorsal margin of female proctiger in profile with strong constriction in the middle (Figure 4d) ····································································································································································································***C. pyri* (L.)**
  - Paramere in profile lamellar. Dorsal margin of female proctiger in profile cuneate························································································································································································································**3**
3. Parameres with a long, forward-directed tooth and a shorter, inwardly directed tooth at apex; foremargin with a wide lobe (Figure 3a). Dorsal margin of female proctiger in profile with a small hump in the middle, apex blunt or subacute (Figure 4a) ···············································································································································································································***C. bidens* (Šulc)**
  - Paramere and aedeagus different. Dorsal margin of female proctiger in profile concave························································································································································································································**4**
4. Paramere with a forward-directed apical tooth (Figure 3c). Female proctiger often clearly exceeding subgenital plate (Figure 4c) ········································································································································································***C. permixta* Burckhardt & Hodkinson**
  - Paramere with inwardly directed blunt apex. Female proctiger only slightly exceeding subgenital plate·····························································································································································································································**5**
5. Forewings with white veins (Figure 1c). Body smaller (head width: 0.55–0.71 mm; forewing length: 1.38–2.09 mm; ratio of antennal length to head width: 1.12–1.57) ·······················································································································································***C. notata* (Flor)**
  - Forewings with yellow to brown or dark brown veins (Figure 1f). Body larger (head width: 0.60–0.73 mm; forewing length: 1.62–2.54 mm; ratio of antennal length to head width: 1.31–1.79) ··································································································*C. pyricola* (Foerster)*

* The presence of *Cacopsylla pyricola* in Iran remains unconfirmed. Although no reliably verified record currently confirms its occurrence in the country, the species is included in the identification key to facilitate its recognition.

### 3.3. Identification of Iranian Pear Psyllids Based on Molecular Characters

In total, COI sequences from ten *Cacopsylla* species were compared. The COI fragments obtained from two voucher specimens of *C. permixta* were successfully amplified and sequenced, and the resulting sequences were deposited in GenBank for the first time under accession numbers PZ344517 and PZ344518 (Table 2). The COI sequences of the remaining nine species were downloaded from GenBank and included in the comparative analyses (Table 2).

The interspecific K2P distances of the analysed sequences ranged from 11.94 to 22.65% (Supplementary Table S1). The lowest interspecific distance (11.94%) was observed between *C. pyricola* (MK039590–MK039594, MK039635, MT021781 and AF493566) and *C. bidens* (MK039595). The highest divergence (22.65%) occurred between *C. bidens* (KT258657) and *C. burckhardti* (MK039639).

**Figure 5:**
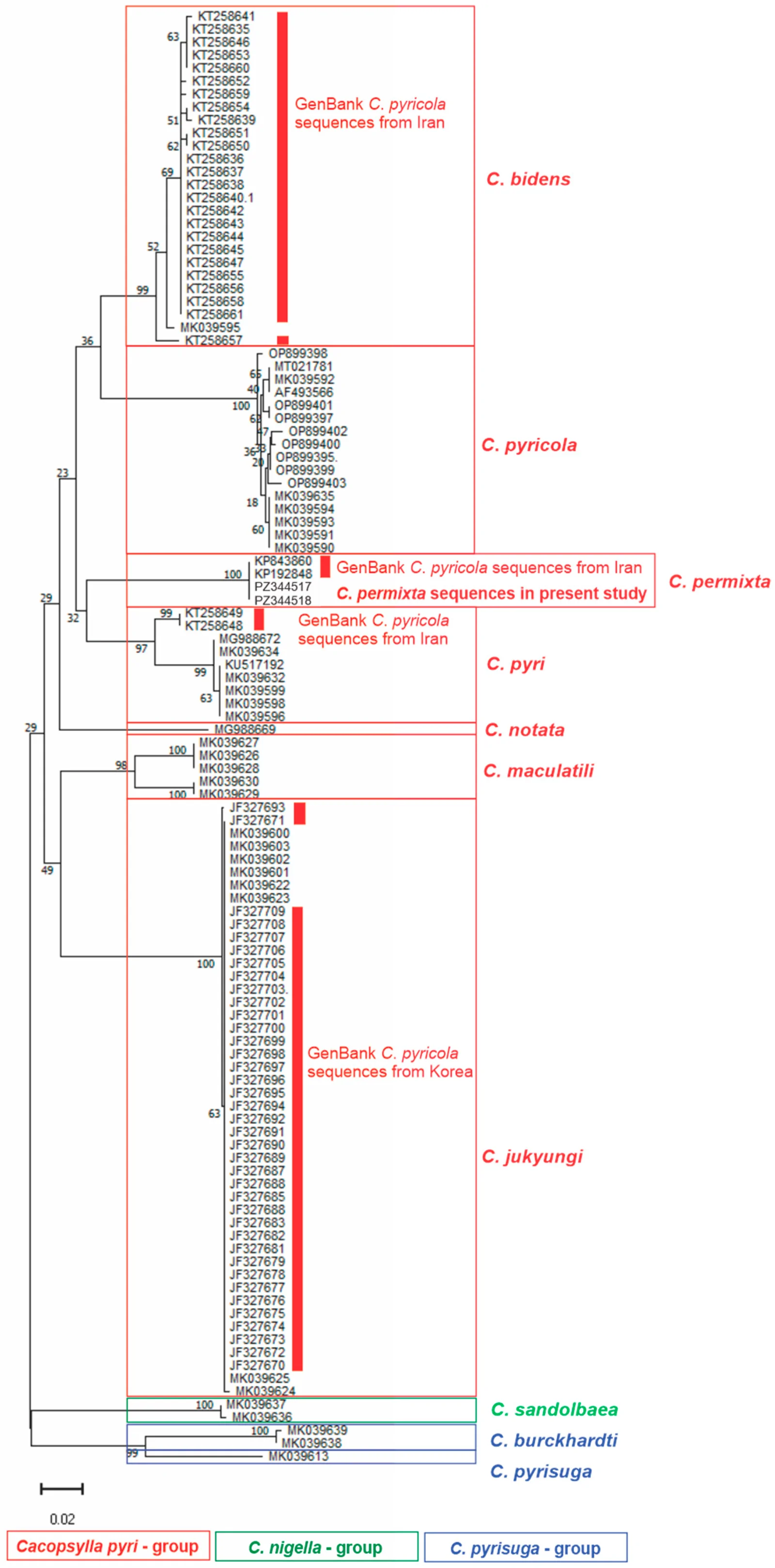
Neighbour-joining tree based on Kimura 2-parameter COI genetic distances between ten *Cacopsylla* spp., associated with pear trees. Bootstrap support values are shown at the branch points and are based on 1000 replicates.

According to our results, all GenBank entries labelled as ‘*Cacopsylla pyricola*’ from Iran (accession numbers KT258635–KT258661, KP192848 and KP843860) were misidentified. Based on our analysis, all sequences submitted by Zendedel (2015) [37] (except KT258648 and KT258649) belong to *C. bidens*. These sequences are genetically close to *C. bidens* from Slovenia [68], differing from the Slovenian sequences by a genetic distance of 1.08%. The two remaining sequences from Zendedel (2015) [37], KT258648 and KT258649, were identified as *C. pyri* and differed from other specimens of this species from the Czech Republic and the UK by genetic distances ranging from 3.84 to 4.12%. A substantial intraspecific genetic divergence was also observed between the Japanese and South Korean lineages of *C. maculatili*, with a genetic distance of 5.61%. The remaining two sequences (KP192848 and KP843860) were also misidentified and, based on our analysis, represent *C. permixta*, showing no genetic divergence from the *C. permixta* specimens sequenced in the present study (Figure 5, Supplementary Table S1).

## 4. Discussion

The accurate identification of species is key to successful biological research [79], as misidentifications can lead to substantial problems in quarantine, pest management and control measures. DNA barcoding has become a widely used and effective tool for species identification [80] and has been successfully applied to numerous psyllid groups [68,81,82,83,84,85,86,87]. This method is particularly valuable for distinguishing taxa that lack distinct morphological differences or that exhibit seasonal or sexual dimorphism [80]. However, a major challenge in DNA barcoding studies is the misidentification of the specimens used to generate reference sequences. Therefore, it is crucial that new entries submitted to public databases such as BOLD and GenBank are accurately identified.

In pear psyllids, misidentifications are especially frequent because several species of the *Cacopsylla pyri*-group are morphologically similar, occur on the same host plants, and show seasonal dimorphism [12]. In addition, some records appear to have been based mainly on the occurrence of specimens in pear orchards rather than on a detailed examination of morphological characters. The newly generated COI sequences from morphologically identified specimens of *C. permixta* are particularly important, because they provide a reliable reference for this species and demonstrate that KP192848 and KP843860, previously labelled as *C. pyricola* in GenBank, were misidentified and actually belong to *C. permixta*.

In the present study, the sequences KT258648 and KT258649, which clustered within *C. pyri*, showed K2P distances of 3.84–4.12% from the other available *C. pyri* sequences. Similarly, the Japanese and South Korean lineages of *C. maculatili* exhibited substantial genetic divergence in the COI gene, with a K2P distance of 5.61% between the two lineages. Although these values exceed the approximately 3% threshold proposed for intraspecific divergence in pear psyllids, such levels of divergence have also been documented in *C. maculatili*. Cho et al. (2020) reported a maximum intraspecific divergence of 5.9% in *C. maculatili* [68]. Percy (2003), in her study of legume-feeding psyllids from the Canary Islands, suggested that high levels of intraspecific mitochondrial divergence may be associated with geographic population structure and historical patterns of dispersal and colonization [88]. Therefore, the relatively high COI divergence observed in the present study should not, by itself, be interpreted as evidence of species-level differentiation. The strong bootstrap support for the placement of KT258648 and KT258649 within the *C. pyri* clade, the clustering of the Japanese and South Korean *C. maculatili* lineages within the *C. maculatili* clade, and their geographic distribution are more consistent with pronounced intraspecific genetic differentiation. Nevertheless, additional independent evidence, particularly from nuclear markers and morphological characters, would be necessary to determine whether these divergent lineages represent geographically structured populations or distinct taxa.

In our study, we also found strong congruence among the molecular, morphological and biological evidence supporting the delimitation of the ten pear psyllid species. The highest genetic distances involved *C. burckhardti* and *C. pyrisuga* relative to the remaining taxa, reflecting their distinct morphology and biology. In accordance with the molecular results, *C. burckhardti* and *C. pyrisuga* differ from the other analysed species in having longer forewings (>3.0 mm), the absence of an apical brown patch on the clavus, and parameres that are relatively long in relation to the length of the distal segment of the aedeagus. The two species also differ biologically from the other species examined here. Members of the *Cacopsylla pyri*-group (seven species: *C. bidens*, *C. jukyungi*, *C. maculatili*, *C. notata*, *C. permixta*, *C. pyri* and *C. pyricola*) and the *C. nigella*-group (*C. sandolbaea*) do not migrate to conifers, overwinter on their host plants, and produce two or more generations per year. In contrast, *C. burckhardti* and *C. pyrisuga* migrate to conifers to overwinter and are univoltine [6,15,16]. Furthermore, *C. sandolbaea* was placed in a separate group. Morphologically, this species is distinct from the other analysed *Cacopsylla* species, having an almost entirely dark brown to black body and forewing membrane, with very dense surface spinules covering the entire membrane up to the veins. These morphological differences are also reflected in its molecular divergence from the remaining taxa. By contrast, the most similar species, both morphologically and molecularly, were *C. bidens* and *C. pyricola*.

In the faunistic literature, *C. pyricola* was reported from Iran by Ossiannilsson (1992) [51] and Burckhardt and Lauterer (1993) [28]. The latter record was considered doubtful and was based on only two females. The species was also reported by Zendedel (2015) [37] based on GenBank information, but without confirmation through morphological identification. The identifications reported by Ahmadi et al. (2012) [33] and Ezzati et al. (2017) [42] are also doubtful, as there is no evidence that they were morphologically confirmed (Table 1). At present, there is no confirmed evidence for the occurrence of *C. pyricola* in Iran. Its presence remains possible, but requires verification using reliably identified voucher specimens, preferably supported by COI sequences.

Taken together, our results show that COI barcoding is a generally useful tool for identifying and distinguishing pear psyllid species, particularly when combined with diagnostic morphological characters. However, the present study also demonstrates that GenBank entries should not be used uncritically for species identification, especially in taxonomically difficult groups. Public barcode databases are valuable only when sequences are linked to correctly identified voucher specimens. To improve our understanding of pear psyllids and to ensure effective management of these pests, their distributions, host plant relationships and biology need to be determined more precisely. Moreover, in Iran, a phytoplasma has been detected in infected pear trees [9], but no psyllid has yet been identified as its vector. Further research is therefore needed to determine whether *C. bidens*, *C. permixta* or *C. pyri* can act as vectors in Iran.

## 5. Conclusions

Based on the examination of COI sequences deposited in GenBank, we suggest that previous records of *Cacopsylla pyricola* from Iran are currently unconfirmed and likely represent misidentifications of other members of the *C. pyri***-**group, particularly *C. bidens*, *C. permixta* or *C. pyri*, all of which occur widely in pear-producing areas of Iran.

These misidentifications likely result from three main factors: (1) a tendency to apply the names *C. pyri* or *C. pyricola* to any psyllid found in pear orchards without confirming its identity using morphological and/or molecular characters, (2) reliance on earlier studies that themselves contained misidentified material, and (3) errors in GenBank entries, including several sequences that were incorrectly attributed to *C. pyricola*.

We therefore strongly recommend that researchers consult expert taxonomists when accurate species-level identification is required, particularly for economically important pest species. For taxonomically problematic taxa, reliance on GenBank entries alone should be avoided.

## Figures and Tables

**Table 1:** Literature and online references reporting *Cacopsylla pyricola* or *Psylla pyricola* from Iran, with the possible identities of specimens suspected to have been misidentified. “Pre 1986” and “post 1986” indicate references published before and after Burckhardt & Hodkinson (1986) [12], respectively.

Reference	Locality	Possible Identity of Specimens Reported as *C. pyricola*
**Pre 1986**		
Farahbakhsh 1961 [23]	Tehran, Caspian Sea area	*C. bidens*, *C. permixta* or *C. pyri*
Davatchi & Esmaili 1966 [24]	Karaj	*C. bidens*, *C. permixta* or *C. pyri*
Radjabi &Behechti 1975 [25]	Iran (without details)	*C. bidens*, *C. permixta* or *C. pyri*
**Post 1986**		
Davoodi 1986 [26]	Tehran	*C. bidens*, *C. permixta* or *C. pyri*
Davoudi &Farzaneh 1991 [27]	Tehran	*C. bidens*, *C. permixta* or *C. pyri*
Burckhardt &Lauterer 1993 [28]	Elborz, Tehran	*C. bidens*, *C. permixta* or *C. pyri*
Heydari Alizadeh &Mossadegh 1997 [29]	Karaj	*C. bidens*, *C. permixta* or *C. pyri*
Jooyandeh et al., 2010 [30]	Tehran	*C. bidens*, *C. permixta* or *C. pyri*
Bayati et al., 2011 [31]	Tehran	*C. bidens*, *C. permixta* or *C. pyri*
Kolyaee 2011 [32]	Tehran	*C. bidens*, *C. permixta* or *C. pyri*
Ahmadi et al., 2012 [33]	East Azerbaijan	*C. bidens*, *C. permixta* or *C. pyri*
Emami &Taheri 2012 [34]	Isfahan	*C. bidens*, *C. permixta* or *C. pyri*
Emami et al., 2014a [35]	Isfahan	*C. bidens*, *C. permixta* or *C. pyri*
Emami et al., 2014b [36]	Isfahan	*C. bidens*, *C. permixta* or *C. pyri*
Zendedel 2015 [37]	Razavi Khorasan, South Khorasan, Isfahan, East Azer-baijan, West Azerbaijan	*C. bidens*, *C. permixta* or *C. pyri*
Ardestanirostami et al., 2016 [38]	Karaj	*C. bidens*, *C. permixta* or *C. pyri*
Zendedel et al., 2016 [39]	Razavi Khorasan, South Khorasan, Isfahan, East Azerbaijan, West Azerbaijan	*C. bidens*, *C. permixta* or *C. pyri*
Emami 2017 [40]	Isfahan	*C. bidens*, *C. permixta* or *C. pyri*
Emami et al., 2017 [41]	Isfahan	*C. bidens*, *C. permixta* or *C. pyri*
Ezzati et al., 2017 [42]	Kurdistan	*C. bidens*, *C. permixta* or *C. pyri*
Sherafat et al., 2018 [43]	Fars, Razavi Khorasan	*C. bidens*, *C. permixta* or *C. pyri*
Emami 2019 [44]	Isfahan	*C. bidens*, *C. permixta* or *C. pyri*
Hassanzadeh-Avval et al., 2020 [45]	Razavi Khorasan	*C. bidens*, *C. permixta* or *C. pyri*
Fathi 2023 [46]	East Azerbaijan	*C. bidens*, *C. permixta* or *C. pyri*
Latifian et al., 2023 [47]	Karaj	*C. bidens*, *C. permixta* or *C. pyri*
Emami 2025 [48]	Isfahan	*C. bidens*, *C. permixta* or *C. pyri*
**GenBank (Accession No.)**		
KT258635– KT258636	Isfahan	*C. bidens*
KT258637–KT258640	Razavi Khorasan, Ferdous	*C. bidens*
KT258641–KT258644	Razavi Khorasan, Mashhad	*C. bidens*
KT258645–KT258647	Razavi Khorasan, Kalat	*C. bidens*
KT258648–KT258649	East Azerbaijan, Urmia	*C. pyri*
KT258650–KT258651	East Azerbaijan, Urmia	*C. bidens*
KT258652–KT258655	Razavi Khorasan, Shandiz	*C. bidens*
KT258656–KT258658	East Azerbaijan, Marand	*C. bidens*
KT258659–KT258661	Razavi Khorasan, Zoshk	*C. bidens*
KP192848	Kerman	*C. permixta*
KP843860	Kerman	*C. permixta*
**Secondary references**		
Gentry 1965 [49]	Iran (without details)	*C. bidens*, *C. permixta* or *C. pyri*
Behdad 1991 [50]	Iran (without details)	*C. bidens*, *C. permixta* or *C. pyri*
Ossiannilsson 1992 [51]	Iran (without details)	*C. bidens*, *C. permixta* or *C. pyri*
Modarres Awal 2002 [52]	Iran (without details)	*C. bidens*, *C. permixta* or *C. pyri*
Lotfalizadeh &Gharali 2008 [53]	Iran (without details)	*C. bidens*, *C. permixta* or *C. pyri*
Askari et al., 2009 [54]	Iran (without details)	*C. bidens*, *C. permixta* or *C. pyri*
Ghahari et al., 2010 [55]	Iran (without details)	*C. bidens*, *C. permixta* or *C. pyri*
Fallahzadeh &Japoshvili 2010 [56]	Iran (without details)	*C. bidens*, *C. permixta* or *C. pyri*
Khaghaninia et al., 2010 [57]	Iran (without details)	*C. bidens*, *C. permixta* or *C. pyri*
Moravvej et al., 2016 [58]	Iran (without details)	*C. bidens*, *C. permixta* or *C. pyri*
Biranvand et al., 2017 [51,59]	Iran (without details)	*C. bidens*, *C. permixta* or *C. pyri*
Hemmati et al., 2021 [60]	Iran (without details)	*C. bidens*, *C. permixta* or *C. pyri*
Sinha et al., 2022 [61]	Iran (without details)	*C. bidens*, *C. permixta* or *C. pyri*
Biranvand et al., 2024 [62]	Iran (without details)	*C. bidens*, *C. permixta* or *C. pyri*
**websites**		
https://mihankesht.ir, accessed on 27 August 2026	Iran (without details)	*C. bidens*, *C. permixta* or *C. pyri*
https://agripars.ir, accessed on 27 August 2026	Iran (without details)	*C. bidens*, *C. permixta* or *C. pyri*
https://bartarnahal.ir, accessed on 27 August 2026	Iran (without details)	*C. bidens*, *C. permixta* or *C. pyri*
https://lib.salamkeshavarzco.ir, accessed on 27 August 2026	Iran (without details)	*C. bidens*, *C. permixta* or *C. pyri*
https://fartakabizendehrood.ir, accessed on 27 August 2026	Iran (without details)	*C. bidens*, *C. permixta* or *C. pyri*
https://narenjspadana.ir, accessed on 27 August 2026	Iran (without details)	*C. bidens*, *C. permixta* or *C. pyri*
https://kavoshkimia.com, accessed on 27 August 2026	Iran (without details)	*C. bidens*, *C. permixta* or *C. pyri*
https://taranomesabz.ir, accessed on 27 August 2026	Iran (without details)	*C. bidens*, *C. permixta* or *C. pyri*

**Table 2:** Accession numbers of COI sequences of pear psyllids (*Cacopsylla* spp.) analysed in the present study. The table compares species names under which the sequences were originally deposited in GenBank with identifications proposed by Cho et al. (2020) [68] and with the corrected identifications established in the present study. Accession numbers generated here for the first time are in bold and correspond to the COI sequences of true *Cacopsylla permixta*.

Accession Number in GenBank	Species Name in GenBank	Country	Reference	ID in Cho et al., 2020	ID in This Study
MK039595	*C. bidens*	Slovenia	Cho et al., 2020 [68]	-	-
MK039638–MK039639	*C. burckhardti*	South Korea	Cho et al., 2020 [68]	-	-
MK039614–MK039617	*C. burckhardti*	South Korea	Cho et al., 2020 [68]	-	-
MK039622–MK039625	*C. jukyungi*	South Korea	Cho et al., 2020 [68]	-	-
MK039600–MK039603	*C. jukyungi*	South Korea	Cho et al., 2020 [68]	-	-
MK039629–MK039630	*C. maculatili*	Japan	Cho et al., 2020 [68]	-	-
MK039626–MK039628	*C. maculatili*	South Korea	Cho et al., 2020 [68]	-	-
MK039604–MK039607	*C. maculatili*	South Korea	Cho et al., 2020 [68]	-	-
MK039608–MK039609	*C. maculatili*	Japan	Cho et al., 2020 [68]	-	-
MG988669	*C. notata*	Bulgaria	Percy et al., 2018 [72]	-	-
**PZ344517**	***C*** **. *permixta***	**Iran**	**Present study**	**-**	***C*** **. *permixta***
**PZ344518**	***C*** **. *permixta***	**Iran**	**Present study**	**-**	***C*** **. *permixta***
MK039632–MK039634	*C. pyri*	Czech Republic	Cho et al., 2020 [68]	-	-
MK039596–MK039599	*C. pyri*	Czech Republic	Cho et al., 2020 [68]	-	-
MG988672	*C. pyri*		Percy et al., 2018 [72]	-	-
KU517192	*C. pyri*	UK	Hodgetts et al., 2016 [73]	-	-
KT258648–KT258649	*C. pyricola*	Iran	Zendedel 2015 [37]	*C. pyri*	*C. pyri*
OP899395–OP899403	*C. pyricola*	Czech Republic	Serbina et al., 2023 [74]	-	-
MT021781	*C. pyricola*	UK	Sumner-Kalkun et al., 2020 [75]	-	-
MK039635	*C. pyricola*	Poland	Cho et al., 2020 [68]	-	-
MK039591–MK039594	*C. pyricola*	Czech Republic	Cho et al., 2020 [68]	-	-
MK039590	*C. pyricola*	Poland	Cho et al., 2020 [68]	-	-
KT258635–KT258661	*C. pyricola*	Iran	Zendedel (2015) [37]	*C. bidens*	*C. bidens*
KP843860	*C. pyricola*	Iran	Zohdi & Hossini, 2015 (unpublished) [76]	*Cacopsylla* sp.	*C. permixta*
KP192848	*C. pyricola*	Iran	Zohdi & Hossini, 2015 (unpublished) [76]	*Cacopsylla* sp.	*C. permixta*
JF327670–JF327709	*C. pyricola*	South Korea	Kang et al., 2012 [77]	*C. jukyungi*	-
AF493566	*C. pyricola*	France	Agustí et al., 2003 [78]	-	-
MK039612–MK039613	*C. pyrisuga*	Czech Republic	Cho et al., 2020 [68]	-	-
MK039636–MK039637	*C. sandolbaea*	South Korea	Cho et al., 2020 [68]	-	-
MK039610–MK039611	*C. sandolbaea*	South Korea	Cho et al., 2020 [68]	-	-

## Data Availability

The COI sequences of *Cacopsylla permixta* generated in this study are available in GenBank under accession numbers PZ344517 and PZ344518.

## Supplementary Materials

The following supporting information can be downloaded at: https://www.mdpi.com/article/10.3390/insects17090915/s1. Supplementary Table S1. Interspecific and intraspecific K2P distances (COI gene fragment) of the analysed pear psyllids (see Table 2 for details on accession numbers).

## Abbreviations

The following abbreviations are used in this manuscript:

KGUT	Department of Biodiversity, Institute of Science and High Technology and Environmental Sciences, Graduate University of Advanced Technology, Kerman, Iran
NHMB	Naturhistorisches Museum, Basel, Switzerland
MHNG	Muséum d’histoire naturelle, Geneva, Switzerland.

## References

[1] Plants of the World Online (POWO). https://powo.science.kew.org/.

[2] Silva G.J., Souza T.M., Barbieri R.L., Oliveira A.C. (2014). Origin, domestication, and dispersing of pear (*Pyrus* spp.). Adv. Agric..

[3] Food and Agriculture Organization of the United Nations (FAO). https://www.fao.org/faostat/en/#data/QCL.

[4] Arzani K. (2004). The effect of European pear (*Pyrus communis* L.) and quince (*Cydonia oblonga* L.) seedling rootstocks on growth and performance of some Asian pear (*Pyrus serotina* Rhed.) cultivars. Acta Hortic..

[5] Burckhardt D. (1994). Psylloid pests of temperate and subtropical crop and ornamental plants (Hemiptera, Psylloidea): A review. Trends Agric. Sci. Entomol..

[6] Hodkinson I.D. (2009). Life cycle variation and adaptation in jumping plant lice (Insecta: Hemiptera: Psylloidea): A global synthesis. J. Nat. Hist..

[7] Jarausch B., Tedeschi R., Sauvion N., Gross J., Jarausch W., Bertaccini A., Weintraub P., Rao G., Mori N. (2019). Psyllid vectors. Phytoplasmas: Plant Pathogenic Bacteria-II: Transmission and Management of Phytoplasma-Associated Diseases.

[8] Štarhová Serbina L., Gajski D., Pafčo B., Zurek L., Malenovský I., Nováková E., Schuler H., Dittmer J. (2022). Microbiome of pear psyllids: A tale about closely related species sharing their endosymbionts. Environ. Microbiol..

[9] Salehi M., Izadpanah K., Taghavi S., Rahimian H. (2008). Characterization of a phytoplasma associated with pear decline in Iran. J. Phytopathol..

[10] Hashemi Tameh M., Bahar M., Zirak L. (2014). Molecular characterization of phytoplasmas related to apple proliferation and aster yellows groups associated with pear decline disease in Iran. J. Phytopathol..

[11] Civolani S., Soroker V., Cooper W.R., Horton D.R. (2023). Diversity, biology, and management of the pear psyllids: A global look. Ann. Entomol. Soc. Am..

[12] Burckhardt D., Hodkinson I.D. (1986). A revision of the west Palaearctic pear psyllids (Hemiptera: Psyllidae). Bull. Entomol. Res..

[13] Yang M.-M., Huang J.-H., Li F. (2004). A new record of *Cacopsylla* species (Hemiptera: Psyllidae) from pear orchards in Taiwan. Formos. Entomol..

[14] Luo X., Li F., Ma Y., Cai W. (2012). A revision of Chinese pear psyllids (Hemiptera: Psylloidea) associated with *Pyrus ussuriensis*. Zootaxa.

[15] Cho G., Burckhardt D., Inoue H., Lee S. (2017). Systematics of the east Palaearctic pear psyllids (Hemiptera: Psylloidea) with particular focus on the Japanese and Korean fauna. Zootaxa.

[16] Lauterer P. (1999). Results of the investigations on Hemiptera in Moravia, made by the Moravian Museum (Psylloidea 2). Acta Musei Morav. Sci. Biol..

[17] Bastin S., Burckhardt D., Reyes-Betancort J.A., Hernández-Suárez E., Ouvrard D. (2023). A review of the jumping plant-lice (Hemiptera: Psylloidea) of the Canary Islands, with descriptions of two new genera and sixteen new species. Zootaxa.

[18] Folmer O., Black M., Hoeh W., Lutz R., Vrijenhoek R. (1994). DNA primers for amplification of mitochondrial cytochrome c oxidase subunit I from diverse metazoan invertebrates. Mol. Mar. Biol. Biotechnol..

[19] Bastin S., Percy D.M., Siverio F. (2023b). Establishing reliable DNA barcoding primers for jumping plant lice (Psylloidea, Hemiptera). BMC Res. Notes.

[20] Srivathsan A., Feng V., Suárez D., Emerson B., Meier R. (2024). Rapid species discovery and identification with real-time barcoding facilitated by ONTbarcoder 2.0 and Oxford Nanopore R10. 4. Cladistics.

[21] Srivathsan A., Lee L., Katoh K., Hartop E., Narayanan Kutty S., Wong J., Yeo D., Meier R. (2021). ONTbarcoder and MinION barcodes aid Biodiversity discovery and identification by everyone, for everyone. BMC Biol..

[22] Tamura K., Stecher G., Kumar S. (2021). MEGA11: Molecular evolutionary genetics analysis version 11. Mol. Biol. Evol..

[23] Farahbakhsh G. (1961). A Checklist of Economically Important Insects and Other Enemies of Plants and Agricultural Products in Iran.

[24] Davatchi A., Esmaili M. (1966). *Psylla pyricola* Forster. Entomol. Phytopathol. Appl..

[25] Radjabi G., Behechti N. (1975). Bio-ecological studies and control of *Psylla pyricola* Foerster (Hom. Psyllidae) in Esfahan 1971–1973. Entomol. Phytopathol. Appl..

[26] Davoodi Z. (1986). Pear psylla in Tehran Province. Entomol. Phytopathol. Appl..

[27] Davoudi Z., Farzaneh A. Some field trials on winter sprays against *Psylla pyricola* and *Parlatoria oleae*. Proceedings of the 10th Iranian Plant Protection Congress.

[28] Burckhardt D., Lauterer P. (1993). The jumping plant-lice of Iran (Homoptera, Psylloidea). Rev. Suisse Zool..

[29] Heydari Alizadeh B., Mossadegh M. (1997). Honeydew producing insects in Tehran Province, Iran. Plant Prot. (Sci. J. Agric.).

[30] Jooyandeh A., Jalaeian M., Sedigh E. Study of effectiveness of common green lacewing *Chrysoperla carnea* (Neu.: Chrysopidae) in control of pear psylla *Cacopsylla* (=*Psylla*) *pyricola* (Hem.: Psyllidae). Proceedings of the 19th Iranian Plant Protection Congress.

[31] Bayati M., Arzani K., Moharramipour S. (2011). Evaluation of resistance of asian pear cultivars to pear psylla (*Cacopsylla pyricola* L.) under Tehran environmental conditions. Iran. J. Hortic. Sci. Technol..

[32] Kolyaee R. (2011). Investigation of the Effect of Diflubenzuron (Dimillin SC48%) for Controlling Pear Psylla.

[33] Ahmadi A., Mehrvar A., Lotfalizadeh H., Gharekhani G., Burckhardt D. (2012). Determination of the species diversity of Psylloidea superfamily (Hemiptera) in East-Azarbaijan Province. J. Sustain. Agric. Prod. Sci..

[34] Emami M., Taheri M. (2012). Study on developmental threshold and thermal constant of *Anthocoris nemoralis* Fabricius, a predator of pear psylla. Iran. J. Biol..

[35] Emami M.S., Shishehbor P., Karimzadeh Esfahani J. (2014). Functional response of *Anthocoris nemoralis* (Hemiptera: Anthocoridae) to the pear psylla, *Cacopsylla pyricola* (Hemiptera: Psyllidae): Effect of pear varieties. J. Crop Prot..

[36] Emami M.S., Shishehbor P., Karimzadeh J. (2014). The influences of plant resistance on predation rate of *Anthocoris nemoralis* (Fabricius) on *Cacopsylla pyricola* (Förster). Arch. Phytopathol. Plant Prot..

[37] Zendedel A. (2015). Study on Fauna of Psyllids in Khorasan-e-Razavi Province and Molecular Study of Different Populations of Pear Psylla, *Cacopsylla pyricola* (Hemiptera: Psylloidea). Master’s Thesis.

[38] Ardestanirostami H., Sheikhigarjan A., Arbab A., Javadzadeh M. (2016). Control of pear psylla, *Cacopsylla pyricola* by trunk injection of azadirachtin and complete fertilizer. Iran. J. Plant Prot. Sci..

[39] Zendedel A., Burckhardt D., Fekrat L., Manzari S., Namaghi H.S. (2016). An annotated checklist of the jumping plant-lice (Hemiptera: Psylloidea) of Iran. J. Entomol. Res. Soc..

[40] Emami M.S. (2017). The effects of plant cover on population of pear psylla (*Cacopsylla pyricola*) and its predators. Acta Agric. Slov..

[41] Emami M.S., Karimzadeh J., Shishehbor P. (2017). Investigating the effect of pear variety on the population equilibrium level of pear psylla, *Cacopsylla pyricola* (Foerster), in field. J. Plant Prot..

[42] Ezzati M., Mehrvar A., Ahmadi Z. (2017). Faunistic study of the superfamily Psylloidea (Hemiptera). IAU Entomol. Res. J..

[43] Sherafat S., Salehi M., Salari M., Rastegari N. (2017). Detection and identification of the phytoplasma associated with pear decline disease and the pear psylla, *Cacopsylla pyricola*, in Fars and Khorasan-e-Razavi Provinces. J. Plant Pathol..

[44] Emami M.S. (2019). Field evaluation of the relative susceptibility of six pear varieties to the pear psylla (*Cacopsylla pyricola* (Foerster, 1848)). Acta Agric. Slov..

[45] Hassanzadeh-Avval M., Sadeghi-Namaghi H., Fekrat L. (2020). Molecular and morphological identification of *Anthocoris* spp. (Hemiptera: Anthocoridae) predators of three economically important psyllid species in Razavi Khorasan Province, northeastern Iran. Biologia.

[46] Fathi S. (2023). The effect of cultivation of different cover crops in pear orchards in population regulation of pear psylla, *Cacopsylla pyricola*. Plant Pest Res..

[47] Latifian M., Abdollahi H., Ghaemi R. (2023). Antixenosis resistance of pear cultivars to pear psylla [*Cacopsylla pyricola* (Foerster)] under the environmental conditions of Karaj in Iran. Seed Plant J..

[48] Emami M.S. (2025). Pre-bloom control of pear psylla (*Cacopsylla pyricola*) using chemical compounds in combination with yellow sticky traps. Int. J. Pest Manag..

[49] Gentry J.W. (1965). Crop Insects of Northeast Africa-Southwest Asia.

[50] Behdad E. (1991). Pests of Fruit Crop in Iran.

[51] Ossiannilsson F. (1992). The Psylloidea (Homoptera) of Fennoscandia and Denmark.

[52] Modarres Awal M. (2002). List of Agricultural Pests and Their Natural Enemies in Iran.

[53] Lotfalizadeh H., Gharali B. (2008). Pteromalidae (Hymenoptera: Chalcidoidea) of Iran: New records and a preliminary checklist. Entomofauna.

[54] Askari O., Farshbaf Pourabad R., Khaganinia S. (2009). Faunistic study of Heteroptera of Zanjanroud region in Zanjan Province of Iran. Munis Entomol. Zool..

[55] Ghahari H., Huang J., Ostovan H., Rastegar J. (2010). Notes on the Iranian fauna of Pteromalidae (Hymenoptera). Efflatounia.

[56] Fallahzadeh M., Japoshvili G. (2010). Checklist of Iranian encyrtids (Hymenoptera: Chalcidoidea) with descriptions of new species. J. Insect Sci..

[57] Khaghaninia S., Askari O., Farshbaf Pourabad R., Shahim K. (2010). Some additional notes about Heteroptera fauna of Qaradag forests-Iran. Munis Entomol. Zool..

[58] Moravvej S.A., Shishehbor P., Lotfalizadeh H. (2016). A checklist of Chalcidoidea (Insecta: Hymenoptera) of Khuzestan in southwestern Iran. J. Insect Biodivers. Syst..

[59] Biranvand A., Tomaszewska W., Li W., Nicolas V., Shakarami J., Fekrat L., Hesami S. (2017). Review of the tribe Chilocorini Mulsant from Iran (Coleoptera, Coccinellidae). ZooKeys.

[60] Hemmati C., Nikooei M., Al-Subhi A.M., Al-Sadi A.M. (2021). History and current status of phytoplasma diseases in the Middle East. Biology.

[61] Sinha A., Chaudhuri T., Arora L. (2022). Residue free farming of fruit crops. Pharma Innov. J..

[62] Biranvand A., Ceryngier P., Vahedi H., Romasi F., Ghobari H., Nedvĕd O. (2024). *Pachyneuron* (Hymenoptera: Pteromalidae) and its relationship to ladybirds (Coleoptera: Coccinellidae): New data from Iran and review of the literature. Int. J. Trop. Insect Sci..

[63] Esmaeily M., Talebi K., Hosseininaveh V., Nozari J., Burckhardt D., Jackson C.J., Oakeshott J.G. (2022). Insecticide resistance in field populations of the pear psyllids *Cacopsylla permixta* and *Cacopsylla bidens* in Iran. Physiol. Entomol..

[64] Lashkari M., Burckhardt D. (2020). Jumping plant-lice (Hemiptera: Psylloidea) of Kerman, Iran, with the description of one new *Cacopsylla* species. Zootaxa.

[65] Ziaaddini F., Yali M.P., Bozorg-Amirkalaee M. (2022). Foliar spraying of elicitors in pear trees induced resistance to *Cacopsylla bidens*. J. Asia-Pac. Entomol..

[66] Moharum F.A. (2013). Pear psylla *Cacopsylla bidens* (Šulc, 1907): A new pest on pear trees in Egypt (Hemiptera: Psylloidea). Egypt. Acad. J. Biol. Sci..

[67] Akbar S.A., Dar M.A., Mahendiran G., Wachkoo A.A. (2018). The first record of pear psylla *Cacopsylla bidens* (Hemiptera: Psyllidae) from India along with notes on seasonal occurrence and some elements of its biology. Orient. Insects.

[68] Cho G., Malenovský I., Burckhardt D., Inoue H., Lee S. (2020). DNA barcoding of pear psyllids (Hemiptera: Psylloidea: Psyllidae), a tale of continued misidentifications. Bull. Entomol. Res..

[69] Ouvrard D., Burckhardt D., Cocquempot C. (2015). An annotated checklist of the jumping plant-lice (Insecta: Hemiptera: Psylloidea) from the Mercantour National Park, with seven new records for France and one new synonymy. Zoosystema.

[70] Mirkarimi A. (1995). An investigation on the oviposition behavior and ontogeny of *Prionomitus mitratus* Dalm. (Hymenoptera: Encyrtidae), parasitoid of *Psylla pyri* L. (Hemiptera: Psyllidae), in the laboratory. Iran. J. Agric. Sci..

[71] Radjabi G. (1989). Insects Attacking Rosaceous Fruit Trees in Iran, Volume III: Homoptera.

[72] Percy D.M., Crampton-Platt A., Sveinsson S., Lemmon A.R., Lemmon E.M., Ouvrard D., Burckhardt D. (2018). Resolving the psyllid tree of life: Phylogenomic analyses of the superfamily Psylloidea (Hemiptera). Syst. Entomol..

[73] Hodgetts J., Ostojá-Starzewski J.C., Prior T., Lawson R., Hall J., Boonham N. (2016). DNA barcoding for biosecurity: Case studies from the UK plant protection program. Genome.

[74] Štarhová Serbina L., Corretto E., Enciso Garcia J.S., Berta M., Giovanelli T., Dittmer J., Schuler H. (2023). Seasonal wild dance of dual endosymbionts in the pear psyllid *Cacopsylla pyricola* (Hemiptera: Psylloidea). Sci. Rep..

[75] Sumner-Kalkun J., Sjölund M., Arnsdorf Y., Carnegie M., Highet F., Ouvrard D., Greenslade A., Bell J.R., Sigvald R., Kenyon D. (2020). A diagnostic real-time PCR assay for the rapid identification of the tomato-potato psyllid, *Bactericera cockerelli* (Šulc, 1909) and development of a psyllid barcoding database. PLoS ONE.

[76] Zohdi H., Hossini R. Nucleotide sequences deposited in GenBank (Accession numbers: KP843860, KP192848), 2015.

[77] Kang A.R., Baek J.Y., Lee S.H., Cho Y.S., Kim W.S., Han Y.S., Kim I. (2012). Geographic homogeneity and high gene flow of the pear psylla, *Cacopsylla pyricola* (Hemiptera: Psyllidae), detected by mitochondrial COI gene and nuclear ribosomal internal transcribed spacer 2. Anim. Cells Syst..

[78] Agustí N., Unruh T., Welter S. (2003). Detecting *Cacopsylla pyricola* (Hemiptera: Psyllidae) in predator guts using COI mitochondrial markers. Bull. Entomol. Res..

[79] Löbl I., Klausnitzer B., Hartmann M., Krell F.-T. (2023). The silent extinction of species and taxonomists—An appeal to science policymakers and legislators. Diversity.

[80] Hebert P.D., Cywinska A., Ball S.L., DeWaard J.R. (2003). Biological identifications through DNA barcodes. Proc. R. Soc. Lond. B Biol. Sci..

[81] Martoni F., Bulman S., Pitman A., Taylor G., Armstrong K. (2018). DNA barcoding highlights cryptic diversity in the New Zealand Psylloidea (Hemiptera: Sternorrhyncha). Diversity.

[82] Lashkari M., Burckhardt D., Gushki R.S. (2020). Molecular and morphometric identification of pistachio psyllids with niche modeling of *Agonoscena pistaciae* (Hemiptera: Aphalaridae). Bull. Entomol. Res..

[83] Lashkari M., Burckhardt D., Kashef S. (2021). Molecular, morphometric and digital automated identification of three *Diaphorina* species (Hemiptera: Liviidae). Bull. Entomol. Res..

[84] Lashkari M., Mirzaei S., Shamsi G.R. (2019). Molecular study of ash psyllids, *Psyllopsis* (Hemiptera: Liviidae), and their *Wolbachia* endosymbiont. J. Entomol. Soc. Iran.

[85] Pramatarova M., Burckhardt D., Malenovský I., Gjonov I., Schuler H., Štarhová Serbina L. (2024). Unravelling the molecular identity of Bulgarian jumping plant lice of the family Aphalaridae (Hemiptera: Psylloidea). Insects.

[86] Percy D.M., Butterill P.T., Malenovský I. (2016). Three new species of gall-forming psyllids (Hemiptera: Psylloidea) from Papua New Guinea, with new records and notes on related species. J. Nat. Hist..

[87] Percy D.M. (2017). Making the most of your host: The Metrosideros-feeding psyllids (Hemiptera, Psylloidea) of the Hawaiian Islands. ZooKeys.

[88] Percy D.M. (2003). Radiation, diversity, and host–plant interactions among island and continental legume-feeding psyllids. Evolution.

