# Supplementary material for: On the Identity of Pear Psyllids in Iran: A Plea Against the Continued Misuse of Cacopsylla pyricola (Hemiptera: Psylloidea)
Source: Insects. 2026 Sep 1;17(9):915. doi: 10.3390/insects17090915 (PMC13607166; doi:10.3390/insects17090915)
Supplement: Supplementary file 1 [file insects-17-00915-s001.zip › insects-4485849-supplementary.pdf]

Supplementary Table S1. Interspecific and intraspecific K2P distances (COI gene fragment) of the analysed pear psyllids (see Table 2 for details on accession numbers).

		1	2	3	4	5	6	7	8	9	10	11	12	13	14	15	16	17	18	19	20
1	MK039637																				
2	MK039636	0.00																			
3	MK039628	0.17	0.18																		
4	MK039627	0.17	0.18	0.00																	
5	MK039626	0.17	0.18	0.00	0.00																
6	MK039630	0.17	0.17	0.06	0.06	0.06															
7	MK039629	0.17	0.17	0.06	0.06	0.06	0.00														
8	MK039600	0.18	0.19	0.14	0.14	0.14	0.15	0.15													
9	MK039603	0.18	0.19	0.14	0.14	0.14	0.15	0.15	0.00												
10	MK039602	0.18	0.19	0.14	0.14	0.14	0.15	0.15	0.00	0.00											
11	MK039601	0.18	0.19	0.14	0.14	0.14	0.15	0.15	0.00	0.00	0.00										
12	MK039625	0.18	0.19	0.14	0.14	0.14	0.15	0.15	0.00	0.00	0.00	0.00									
13	MK039624	0.19	0.19	0.14	0.14	0.14	0.15	0.15	0.00	0.00	0.00	0.00	0.00								
14	MK039622	0.18	0.19	0.14	0.14	0.14	0.15	0.15	0.00	0.00	0.00	0.00	0.00	0.00							
15	MK039623	0.18	0.19	0.14	0.14	0.14	0.15	0.15	0.00	0.00	0.00	0.00	0.00	0.00	0.00						
16	MK039639	0.22	0.22	0.19	0.19	0.19	0.16	0.16	0.21	0.21	0.21	0.21	0.21	0.22	0.21	0.21					
17	MK039638	0.22	0.22	0.18	0.18	0.18	0.16	0.16	0.21	0.21	0.21	0.21	0.21	0.21	0.21	0.21	0.00				
18	MK039595	0.18	0.18	0.16	0.16	0.16	0.15	0.15	0.15	0.15	0.15	0.15	0.15	0.15	0.15	0.15	0.22	0.21			
19	PZ344517	0.19	0.20	0.20	0.20	0.20	0.18	0.18	0.18	0.18	0.18	0.18	0.18	0.19	0.18	0.18	0.20	0.21	0.16		
20	PZ344518	0.19	0.20	0.20	0.20	0.20	0.18	0.18	0.18	0.18	0.18	0.18	0.18	0.19	0.18	0.18	0.20	0.21	0.16	0.00	

(continued on next table)

Table S1. (continued)

		1	2	3	4	5	6	7	8	9	10	11	12	13	14	15	16	17	18	19	20
21	AF493566	0.17	0.17	0.18	0.18	0.18	0.15	0.15	0.20	0.20	0.20	0.20	0.20	0.20	0.20	0.20	0.21	0.21	0.12	0.16	0.16
22	JF327709	0.18	0.19	0.14	0.14	0.14	0.15	0.15	0.00	0.00	0.00	0.00	0.00	0.00	0.00	0.00	0.21	0.21	0.15	0.18	0.18
23	JF327708	0.18	0.19	0.14	0.14	0.14	0.15	0.15	0.00	0.00	0.00	0.00	0.00	0.00	0.00	0.00	0.21	0.21	0.15	0.18	0.18
24	JF327707	0.18	0.19	0.14	0.14	0.14	0.15	0.15	0.00	0.00	0.00	0.00	0.00	0.00	0.00	0.00	0.21	0.21	0.15	0.18	0.18
25	JF327706	0.18	0.19	0.14	0.14	0.14	0.15	0.15	0.00	0.00	0.00	0.00	0.00	0.00	0.00	0.00	0.21	0.21	0.15	0.18	0.18
26	JF327705	0.18	0.19	0.14	0.14	0.14	0.15	0.15	0.00	0.00	0.00	0.00	0.00	0.00	0.00	0.00	0.21	0.21	0.15	0.18	0.18
27	JF327704	0.18	0.19	0.14	0.14	0.14	0.15	0.15	0.00	0.00	0.00	0.00	0.00	0.00	0.00	0.00	0.21	0.21	0.15	0.18	0.18
28	JF327703	0.18	0.19	0.14	0.14	0.14	0.15	0.15	0.00	0.00	0.00	0.00	0.00	0.00	0.00	0.00	0.21	0.21	0.15	0.18	0.18
29	JF327702	0.18	0.19	0.14	0.14	0.14	0.15	0.15	0.00	0.00	0.00	0.00	0.00	0.00	0.00	0.00	0.21	0.21	0.15	0.18	0.18
30	JF327701	0.18	0.19	0.14	0.14	0.14	0.15	0.15	0.00	0.00	0.00	0.00	0.00	0.00	0.00	0.00	0.21	0.21	0.15	0.18	0.18
31	JF327700	0.18	0.19	0.14	0.14	0.14	0.15	0.15	0.00	0.00	0.00	0.00	0.00	0.00	0.00	0.00	0.21	0.21	0.15	0.18	0.18
32	JF327699	0.18	0.19	0.14	0.14	0.14	0.15	0.15	0.00	0.00	0.00	0.00	0.00	0.00	0.00	0.00	0.21	0.21	0.15	0.18	0.18
33	JF327698	0.18	0.19	0.14	0.14	0.14	0.15	0.15	0.00	0.00	0.00	0.00	0.00	0.00	0.00	0.00	0.21	0.21	0.15	0.18	0.18
34	JF327697	0.18	0.19	0.14	0.14	0.14	0.15	0.15	0.00	0.00	0.00	0.00	0.00	0.00	0.00	0.00	0.21	0.21	0.15	0.18	0.18
35	JF327696	0.18	0.19	0.14	0.14	0.14	0.15	0.15	0.00	0.00	0.00	0.00	0.00	0.00	0.00	0.00	0.21	0.21	0.15	0.18	0.18
36	JF327695	0.18	0.19	0.14	0.14	0.14	0.15	0.15	0.00	0.00	0.00	0.00	0.00	0.00	0.00	0.00	0.21	0.21	0.15	0.18	0.18
37	JF327694	0.18	0.19	0.14	0.14	0.14	0.15	0.15	0.00	0.00	0.00	0.00	0.00	0.00	0.00	0.00	0.21	0.21	0.15	0.18	0.18
38	JF327693	0.18	0.19	0.14	0.14	0.14	0.14	0.14	0.00	0.00	0.00	0.00	0.00	0.01	0.00	0.00	0.21	0.21	0.14	0.18	0.18
39	JF327692	0.18	0.19	0.14	0.14	0.14	0.15	0.15	0.00	0.00	0.00	0.00	0.00	0.00	0.00	0.00	0.21	0.21	0.15	0.18	0.18
40	JF327691	0.18	0.19	0.14	0.14	0.14	0.15	0.15	0.00	0.00	0.00	0.00	0.00	0.00	0.00	0.00	0.21	0.21	0.15	0.18	0.18

(continued on next table)

Table S1. (continued)

		21	22	23	24	25	26	27	28	29	30	31	32	33	34	35	36	37	38	39	40
21	AF493566	0.20																			
22	JF327709	0.20	0.00																		
23	JF327708	0.20	0.00	0.00																	
24	JF327707	0.20	0.00	0.00	0.00																
25	JF327706	0.20	0.00	0.00	0.00	0.00															
26	JF327705	0.20	0.00	0.00	0.00	0.00	0.00														
27	JF327704	0.20	0.00	0.00	0.00	0.00	0.00	0.00													
28	JF327703	0.20	0.00	0.00	0.00	0.00	0.00	0.00	0.00												
29	JF327702	0.20	0.00	0.00	0.00	0.00	0.00	0.00	0.00	0.00											
30	JF327701	0.20	0.00	0.00	0.00	0.00	0.00	0.00	0.00	0.00	0.00										
31	JF327700	0.20	0.00	0.00	0.00	0.00	0.00	0.00	0.00	0.00	0.00	0.00									
32	JF327699	0.20	0.00	0.00	0.00	0.00	0.00	0.00	0.00	0.00	0.00	0.00	0.00								
33	JF327698	0.20	0.00	0.00	0.00	0.00	0.00	0.00	0.00	0.00	0.00	0.00	0.00	0.00							
34	JF327697	0.20	0.00	0.00	0.00	0.00	0.00	0.00	0.00	0.00	0.00	0.00	0.00	0.00	0.00						
35	JF327696	0.20	0.00	0.00	0.00	0.00	0.00	0.00	0.00	0.00	0.00	0.00	0.00	0.00	0.00	0.00					
36	JF327695	0.20	0.00	0.00	0.00	0.00	0.00	0.00	0.00	0.00	0.00	0.00	0.00	0.00	0.00	0.00	0.00				
37	JF327694	0.20	0.00	0.00	0.00	0.00	0.00	0.00	0.00	0.00	0.00	0.00	0.00	0.00	0.00	0.00	0.00	0.00			
38	JF327693	0.20	0.00	0.00	0.00	0.00	0.00	0.00	0.00	0.00	0.00	0.00	0.00	0.00	0.00	0.00	0.00	0.00	0.00		
39	JF327692	0.20	0.00	0.00	0.00	0.00	0.00	0.00	0.00	0.00	0.00	0.00	0.00	0.00	0.00	0.00	0.00	0.00	0.00	0.00	
40	JF327691	0.20	0.00	0.00	0.00	0.00	0.00	0.00	0.00	0.00	0.00	0.00	0.00	0.00	0.00	0.00	0.00	0.00	0.00	0.00	0.00

(continued on next table)

Table S1. (continued)

		1	2	3	4	5	6	7	8	9	10	11	12	13	14	15	16	17	18	19	20
41	JF327690	0.18	0.19	0.14	0.14	0.14	0.15	0.15	0.00	0.00	0.00	0.00	0.00	0.00	0.00	0.00	0.21	0.21	0.15	0.18	0.18
42	JF327689	0.18	0.19	0.14	0.14	0.14	0.15	0.15	0.00	0.00	0.00	0.00	0.00	0.00	0.00	0.00	0.21	0.21	0.15	0.18	0.18
43	JF327687	0.18	0.19	0.14	0.14	0.14	0.15	0.15	0.00	0.00	0.00	0.00	0.00	0.00	0.00	0.00	0.21	0.21	0.15	0.18	0.18
44	JF327688	0.18	0.19	0.14	0.14	0.14	0.15	0.15	0.00	0.00	0.00	0.00	0.00	0.00	0.00	0.00	0.21	0.21	0.15	0.18	0.18
45	JF327685	0.18	0.19	0.14	0.14	0.14	0.15	0.15	0.00	0.00	0.00	0.00	0.00	0.00	0.00	0.00	0.21	0.21	0.15	0.18	0.18
46	JF327688	0.18	0.19	0.14	0.14	0.14	0.15	0.15	0.00	0.00	0.00	0.00	0.00	0.00	0.00	0.00	0.21	0.21	0.15	0.18	0.18
47	JF327683	0.18	0.19	0.14	0.14	0.14	0.15	0.15	0.00	0.00	0.00	0.00	0.00	0.00	0.00	0.00	0.21	0.21	0.15	0.18	0.18
48	JF327682	0.18	0.19	0.14	0.14	0.14	0.15	0.15	0.00	0.00	0.00	0.00	0.00	0.00	0.00	0.00	0.21	0.21	0.15	0.18	0.18
49	JF327681	0.18	0.19	0.14	0.14	0.14	0.15	0.15	0.00	0.00	0.00	0.00	0.00	0.00	0.00	0.00	0.21	0.21	0.15	0.18	0.18
50	JF327679	0.18	0.19	0.14	0.14	0.14	0.15	0.15	0.00	0.00	0.00	0.00	0.00	0.00	0.00	0.00	0.21	0.21	0.15	0.18	0.18
51	JF327678	0.18	0.19	0.14	0.14	0.14	0.15	0.15	0.00	0.00	0.00	0.00	0.00	0.00	0.00	0.00	0.21	0.21	0.15	0.18	0.18
52	JF327677	0.18	0.19	0.14	0.14	0.14	0.15	0.15	0.00	0.00	0.00	0.00	0.00	0.00	0.00	0.00	0.21	0.21	0.15	0.18	0.18
53	JF327676	0.18	0.19	0.14	0.14	0.14	0.15	0.15	0.00	0.00	0.00	0.00	0.00	0.00	0.00	0.00	0.21	0.21	0.15	0.18	0.18
54	JF327675	0.18	0.19	0.14	0.14	0.14	0.15	0.15	0.00	0.00	0.00	0.00	0.00	0.00	0.00	0.00	0.21	0.21	0.15	0.18	0.18
55	JF327674	0.18	0.19	0.14	0.14	0.14	0.15	0.15	0.00	0.00	0.00	0.00	0.00	0.00	0.00	0.00	0.21	0.21	0.15	0.18	0.18
56	JF327673	0.18	0.19	0.14	0.14	0.14	0.15	0.15	0.00	0.00	0.00	0.00	0.00	0.00	0.00	0.00	0.21	0.21	0.15	0.18	0.18
57	JF327672	0.18	0.19	0.14	0.14	0.14	0.15	0.15	0.00	0.00	0.00	0.00	0.00	0.00	0.00	0.00	0.21	0.21	0.15	0.18	0.18
58	JF327671	0.18	0.19	0.14	0.14	0.14	0.15	0.15	0.00	0.00	0.00	0.00	0.00	0.00	0.00	0.00	0.21	0.21	0.15	0.18	0.18
59	JF327670	0.18	0.19	0.14	0.14	0.14	0.15	0.15	0.00	0.00	0.00	0.00	0.00	0.00	0.00	0.00	0.21	0.21	0.15	0.18	0.18
60	OP899403	0.17	0.17	0.18	0.18	0.18	0.15	0.15	0.20	0.20	0.20	0.20	0.20	0.20	0.20	0.20	0.21	0.21	0.13	0.17	0.17

(continued on next table)

Table S1. (continued)

		21	22	23	24	25	26	27	28	29	30	31	32	33	34	35	36	37	38	39	40
41	JF327690	0.20	0.00	0.00	0.00	0.00	0.00	0.00	0.00	0.00	0.00	0.00	0.00	0.00	0.00	0.00	0.00	0.00	0.00	0.00	0.00
42	JF327689	0.20	0.00	0.00	0.00	0.00	0.00	0.00	0.00	0.00	0.00	0.00	0.00	0.00	0.00	0.00	0.00	0.00	0.00	0.00	0.00
43	JF327687	0.20	0.00	0.00	0.00	0.00	0.00	0.00	0.00	0.00	0.00	0.00	0.00	0.00	0.00	0.00	0.00	0.00	0.00	0.00	0.00
44	JF327688	0.20	0.00	0.00	0.00	0.00	0.00	0.00	0.00	0.00	0.00	0.00	0.00	0.00	0.00	0.00	0.00	0.00	0.00	0.00	0.00
45	JF327685	0.20	0.00	0.00	0.00	0.00	0.00	0.00	0.00	0.00	0.00	0.00	0.00	0.00	0.00	0.00	0.00	0.00	0.00	0.00	0.00
46	JF327688	0.20	0.00	0.00	0.00	0.00	0.00	0.00	0.00	0.00	0.00	0.00	0.00	0.00	0.00	0.00	0.00	0.00	0.00	0.00	0.00
47	JF327683	0.20	0.00	0.00	0.00	0.00	0.00	0.00	0.00	0.00	0.00	0.00	0.00	0.00	0.00	0.00	0.00	0.00	0.00	0.00	0.00
48	JF327682	0.20	0.00	0.00	0.00	0.00	0.00	0.00	0.00	0.00	0.00	0.00	0.00	0.00	0.00	0.00	0.00	0.00	0.00	0.00	0.00
49	JF327681	0.20	0.00	0.00	0.00	0.00	0.00	0.00	0.00	0.00	0.00	0.00	0.00	0.00	0.00	0.00	0.00	0.00	0.00	0.00	0.00
50	JF327679	0.20	0.00	0.00	0.00	0.00	0.00	0.00	0.00	0.00	0.00	0.00	0.00	0.00	0.00	0.00	0.00	0.00	0.00	0.00	0.00
51	JF327678	0.20	0.00	0.00	0.00	0.00	0.00	0.00	0.00	0.00	0.00	0.00	0.00	0.00	0.00	0.00	0.00	0.00	0.00	0.00	0.00
52	JF327677	0.20	0.00	0.00	0.00	0.00	0.00	0.00	0.00	0.00	0.00	0.00	0.00	0.00	0.00	0.00	0.00	0.00	0.00	0.00	0.00
53	JF327676	0.20	0.00	0.00	0.00	0.00	0.00	0.00	0.00	0.00	0.00	0.00	0.00	0.00	0.00	0.00	0.00	0.00	0.00	0.00	0.00
54	JF327675	0.20	0.00	0.00	0.00	0.00	0.00	0.00	0.00	0.00	0.00	0.00	0.00	0.00	0.00	0.00	0.00	0.00	0.00	0.00	0.00
55	JF327674	0.20	0.00	0.00	0.00	0.00	0.00	0.00	0.00	0.00	0.00	0.00	0.00	0.00	0.00	0.00	0.00	0.00	0.00	0.00	0.00
56	JF327673	0.20	0.00	0.00	0.00	0.00	0.00	0.00	0.00	0.00	0.00	0.00	0.00	0.00	0.00	0.00	0.00	0.00	0.00	0.00	0.00
57	JF327672	0.20	0.00	0.00	0.00	0.00	0.00	0.00	0.00	0.00	0.00	0.00	0.00	0.00	0.00	0.00	0.00	0.00	0.00	0.00	0.00
58	JF327671	0.20	0.00	0.00	0.00	0.00	0.00	0.00	0.00	0.00	0.00	0.00	0.00	0.00	0.00	0.00	0.00	0.00	0.00	0.00	0.00
59	JF327670	0.20	0.00	0.00	0.00	0.00	0.00	0.00	0.00	0.00	0.00	0.00	0.00	0.00	0.00	0.00	0.00	0.00	0.00	0.00	0.00
60	OP899403	0.01	0.20	0.20	0.20	0.20	0.20	0.20	0.20	0.20	0.20	0.20	0.20	0.20	0.20	0.20	0.20	0.20	0.20	0.20	0.20

(continued on next table)

Table S1. (continued)

		41	42	43	44	45	46	47	48	49	50	51	52	53	54	55	56	57	58	59	60
41	JF327690																				
42	JF327689	0.00																			
43	JF327687	0.00	0.00																		
44	JF327688	0.00	0.00	0.00																	
45	JF327685	0.00	0.00	0.00	0.00																
46	JF327688	0.00	0.00	0.00	0.00	0.00															
47	JF327683	0.00	0.00	0.00	0.00	0.00	0.00														
48	JF327682	0.00	0.00	0.00	0.00	0.00	0.00	0.00													
49	JF327681	0.00	0.00	0.00	0.00	0.00	0.00	0.00	0.00												
50	JF327679	0.00	0.00	0.00	0.00	0.00	0.00	0.00	0.00	0.00											
51	JF327678	0.00	0.00	0.00	0.00	0.00	0.00	0.00	0.00	0.00	0.00										
52	JF327677	0.00	0.00	0.00	0.00	0.00	0.00	0.00	0.00	0.00	0.00	0.00									
53	JF327676	0.00	0.00	0.00	0.00	0.00	0.00	0.00	0.00	0.00	0.00	0.00	0.00								
54	JF327675	0.00	0.00	0.00	0.00	0.00	0.00	0.00	0.00	0.00	0.00	0.00	0.00	0.00							
55	JF327674	0.00	0.00	0.00	0.00	0.00	0.00	0.00	0.00	0.00	0.00	0.00	0.00	0.00	0.00						
56	JF327673	0.00	0.00	0.00	0.00	0.00	0.00	0.00	0.00	0.00	0.00	0.00	0.00	0.00	0.00	0.00					
57	JF327672	0.00	0.00	0.00	0.00	0.00	0.00	0.00	0.00	0.00	0.00	0.00	0.00	0.00	0.00	0.00	0.00				
58	JF327671	0.00	0.00	0.00	0.00	0.00	0.00	0.00	0.00	0.00	0.00	0.00	0.00	0.00	0.00	0.00	0.00	0.00			
59	JF327670	0.00	0.00	0.00	0.00	0.00	0.00	0.00	0.00	0.00	0.00	0.00	0.00	0.00	0.00	0.00	0.00	0.00	0.00		
60	OP899403	0.00	0.00	0.00	0.00	0.00	0.00	0.00	0.00	0.00	0.00	0.00	0.00	0.00	0.00	0.00	0.00	0.00	0.00	0.00	

(continued on next table)

Table S1. (continued)

		1	2	3	4	5	6	7	8	9	10	11	12	13	14	15	16	17	18	19	20
61	OP899402	0.18	0.18	0.18	0.18	0.18	0.15	0.15	0.20	0.20	0.20	0.20	0.20	0.21	0.20	0.20	0.21	0.20	0.13	0.17	0.17
62	OP899401	0.17	0.17	0.18	0.18	0.18	0.15	0.15	0.20	0.20	0.20	0.20	0.20	0.20	0.20	0.20	0.21	0.20	0.12	0.15	0.15
63	OP899400	0.17	0.18	0.17	0.17	0.17	0.15	0.15	0.20	0.20	0.20	0.20	0.20	0.20	0.20	0.20	0.20	0.20	0.12	0.16	0.16
64	OP899399	0.17	0.17	0.17	0.17	0.17	0.15	0.15	0.20	0.20	0.20	0.20	0.20	0.20	0.20	0.20	0.20	0.20	0.12	0.16	0.16
65	OP899398	0.17	0.17	0.17	0.17	0.17	0.14	0.14	0.19	0.19	0.19	0.19	0.19	0.20	0.19	0.19	0.20	0.19	0.12	0.16	0.16
66	OP899397	0.17	0.17	0.18	0.18	0.18	0.15	0.15	0.20	0.20	0.20	0.20	0.20	0.20	0.20	0.20	0.21	0.20	0.12	0.15	0.15
67	OP899395	0.17	0.17	0.17	0.17	0.17	0.15	0.15	0.20	0.20	0.20	0.20	0.20	0.20	0.20	0.20	0.20	0.20	0.12	0.16	0.16
68	MT021781	0.17	0.17	0.18	0.18	0.18	0.15	0.15	0.20	0.20	0.20	0.20	0.20	0.20	0.20	0.20	0.21	0.21	0.12	0.16	0.16
69	MK039635	0.17	0.17	0.17	0.17	0.17	0.15	0.15	0.20	0.20	0.20	0.20	0.20	0.20	0.20	0.20	0.21	0.20	0.12	0.17	0.17
70	MK039594	0.17	0.17	0.17	0.17	0.17	0.15	0.15	0.20	0.20	0.20	0.20	0.20	0.20	0.20	0.20	0.21	0.20	0.12	0.17	0.17
71	MK039593	0.17	0.17	0.17	0.17	0.17	0.15	0.15	0.20	0.20	0.20	0.20	0.20	0.20	0.20	0.20	0.21	0.20	0.12	0.17	0.17
72	MK039592	0.17	0.17	0.18	0.18	0.18	0.15	0.15	0.20	0.20	0.20	0.20	0.20	0.20	0.20	0.20	0.21	0.21	0.12	0.16	0.16
73	MK039591	0.17	0.17	0.17	0.17	0.17	0.15	0.15	0.20	0.20	0.20	0.20	0.20	0.20	0.20	0.20	0.21	0.20	0.12	0.17	0.17
74	MK039590	0.17	0.17	0.17	0.17	0.17	0.15	0.15	0.20	0.20	0.20	0.20	0.20	0.20	0.20	0.20	0.21	0.20	0.12	0.17	0.17
75	KT258661	0.18	0.18	0.16	0.16	0.16	0.15	0.15	0.15	0.15	0.15	0.15	0.15	0.15	0.15	0.15	0.22	0.22	0.01	0.17	0.17
76	KT258660	0.18	0.19	0.17	0.17	0.17	0.15	0.15	0.15	0.15	0.15	0.15	0.15	0.16	0.15	0.15	0.22	0.22	0.01	0.17	0.17
77	KT258659	0.18	0.18	0.17	0.17	0.17	0.15	0.15	0.15	0.15	0.15	0.15	0.15	0.16	0.15	0.15	0.22	0.22	0.01	0.16	0.16
78	KT258658	0.18	0.18	0.16	0.16	0.16	0.15	0.15	0.15	0.15	0.15	0.15	0.15	0.15	0.15	0.15	0.22	0.22	0.01	0.17	0.17
79	KT258657	0.18	0.18	0.17	0.17	0.17	0.15	0.15	0.15	0.15	0.15	0.15	0.15	0.15	0.15	0.15	0.23	0.22	0.02	0.15	0.15
80	KT258656	0.18	0.18	0.16	0.16	0.16	0.15	0.15	0.15	0.15	0.15	0.15	0.15	0.15	0.15	0.15	0.22	0.22	0.01	0.17	0.17

(continued on next table)

Table S1. (continued)

		21	22	23	24	25	26	27	28	29	30	31	32	33	34	35	36	37	38	39	40
61	OP899402	0.02	0.20	0.20	0.20	0.20	0.20	0.20	0.20	0.20	0.20	0.20	0.20	0.20	0.20	0.20	0.20	0.20	0.20	0.20	0.20
62	OP899401	0.01	0.20	0.20	0.20	0.20	0.20	0.20	0.20	0.20	0.20	0.20	0.20	0.20	0.20	0.20	0.20	0.20	0.20	0.20	0.20
63	OP899400	0.01	0.20	0.20	0.20	0.20	0.20	0.20	0.20	0.20	0.20	0.20	0.20	0.20	0.20	0.20	0.20	0.20	0.20	0.20	0.20
64	OP899399	0.01	0.20	0.20	0.20	0.20	0.20	0.20	0.20	0.20	0.20	0.20	0.20	0.20	0.20	0.20	0.20	0.20	0.20	0.20	0.20
65	OP899398	0.01	0.19	0.19	0.19	0.19	0.19	0.19	0.19	0.19	0.19	0.19	0.19	0.19	0.19	0.19	0.19	0.19	0.19	0.19	0.19
66	OP899397	0.01	0.20	0.20	0.20	0.20	0.20	0.20	0.20	0.20	0.20	0.20	0.20	0.20	0.20	0.20	0.20	0.20	0.20	0.20	0.20
67	OP899395	0.01	0.20	0.20	0.20	0.20	0.20	0.20	0.20	0.20	0.20	0.20	0.20	0.20	0.20	0.20	0.20	0.20	0.20	0.20	0.20
68	MT021781	0.00	0.20	0.20	0.20	0.20	0.20	0.20	0.20	0.20	0.20	0.20	0.20	0.20	0.20	0.20	0.20	0.20	0.20	0.20	0.20
69	MK039635	0.01	0.20	0.20	0.20	0.20	0.20	0.20	0.20	0.20	0.20	0.20	0.20	0.20	0.20	0.20	0.20	0.20	0.20	0.20	0.20
70	MK039594	0.01	0.20	0.20	0.20	0.20	0.20	0.20	0.20	0.20	0.20	0.20	0.20	0.20	0.20	0.20	0.20	0.20	0.20	0.20	0.20
71	MK039593	0.01	0.20	0.20	0.20	0.20	0.20	0.20	0.20	0.20	0.20	0.20	0.20	0.20	0.20	0.20	0.20	0.20	0.20	0.20	0.20
72	MK039592	0.00	0.20	0.20	0.20	0.20	0.20	0.20	0.20	0.20	0.20	0.20	0.20	0.20	0.20	0.20	0.20	0.20	0.20	0.20	0.20
73	MK039591	0.01	0.20	0.20	0.20	0.20	0.20	0.20	0.20	0.20	0.20	0.20	0.20	0.20	0.20	0.20	0.20	0.20	0.20	0.20	0.20
74	MK039590	0.01	0.20	0.20	0.20	0.20	0.20	0.20	0.20	0.20	0.20	0.20	0.20	0.20	0.20	0.20	0.20	0.20	0.20	0.20	0.20
75	KT258661	0.12	0.15	0.15	0.15	0.15	0.15	0.15	0.15	0.15	0.15	0.15	0.15	0.15	0.15	0.15	0.15	0.15	0.15	0.15	0.15
76	KT258660	0.12	0.15	0.15	0.15	0.15	0.15	0.15	0.15	0.15	0.15	0.15	0.15	0.15	0.15	0.15	0.15	0.15	0.15	0.15	0.15
77	KT258659	0.12	0.15	0.15	0.15	0.15	0.15	0.15	0.15	0.15	0.15	0.15	0.15	0.15	0.15	0.15	0.15	0.15	0.15	0.15	0.15
78	KT258658	0.12	0.15	0.15	0.15	0.15	0.15	0.15	0.15	0.15	0.15	0.15	0.15	0.15	0.15	0.15	0.15	0.15	0.15	0.15	0.15
79	KT258657	0.11	0.15	0.15	0.15	0.15	0.15	0.15	0.15	0.15	0.15	0.15	0.15	0.15	0.15	0.15	0.15	0.15	0.15	0.15	0.15
80	KT258656	0.12	0.15	0.15	0.15	0.15	0.15	0.15	0.15	0.15	0.15	0.15	0.15	0.15	0.15	0.15	0.15	0.15	0.15	0.15	0.15

(continued on next table)

Table S1. (continued)

		41	42	43	44	45	46	47	48	49	50	51	52	53	54	55	56	57	58	59	60
61	OP899402	0.20	0.20	0.20	0.20	0.20	0.20	0.20	0.20	0.20	0.20	0.20	0.20	0.20	0.20	0.20	0.20	0.20	0.20	0.20	
62	OP899401	0.20	0.20	0.20	0.20	0.20	0.20	0.20	0.20	0.20	0.20	0.20	0.20	0.20	0.20	0.20	0.20	0.20	0.20	0.20	0.01
63	OP899400	0.20	0.20	0.20	0.20	0.20	0.20	0.20	0.20	0.20	0.20	0.20	0.20	0.20	0.20	0.20	0.20	0.20	0.20	0.20	0.01
64	OP899399	0.20	0.20	0.20	0.20	0.20	0.20	0.20	0.20	0.20	0.20	0.20	0.20	0.20	0.20	0.20	0.20	0.20	0.20	0.20	0.01
65	OP899398	0.20	0.20	0.20	0.20	0.20	0.20	0.20	0.20	0.20	0.20	0.20	0.20	0.20	0.20	0.20	0.20	0.20	0.20	0.20	0.01
66	OP899397	0.19	0.19	0.19	0.19	0.19	0.19	0.19	0.19	0.19	0.19	0.19	0.19	0.19	0.19	0.19	0.19	0.19	0.19	0.19	0.01
67	OP899395	0.20	0.20	0.20	0.20	0.20	0.20	0.20	0.20	0.20	0.20	0.20	0.20	0.20	0.20	0.20	0.20	0.20	0.20	0.20	0.01
68	MT021781	0.20	0.20	0.20	0.20	0.20	0.20	0.20	0.20	0.20	0.20	0.20	0.20	0.20	0.20	0.20	0.20	0.20	0.20	0.20	0.01
69	MK039635	0.20	0.20	0.20	0.20	0.20	0.20	0.20	0.20	0.20	0.20	0.20	0.20	0.20	0.20	0.20	0.20	0.20	0.20	0.20	0.01
70	MK039594	0.20	0.20	0.20	0.20	0.20	0.20	0.20	0.20	0.20	0.20	0.20	0.20	0.20	0.20	0.20	0.20	0.20	0.20	0.20	0.01
71	MK039593	0.20	0.20	0.20	0.20	0.20	0.20	0.20	0.20	0.20	0.20	0.20	0.20	0.20	0.20	0.20	0.20	0.20	0.20	0.20	0.01
72	MK039592	0.20	0.20	0.20	0.20	0.20	0.20	0.20	0.20	0.20	0.20	0.20	0.20	0.20	0.20	0.20	0.20	0.20	0.20	0.20	0.01
73	MK039591	0.20	0.20	0.20	0.20	0.20	0.20	0.20	0.20	0.20	0.20	0.20	0.20	0.20	0.20	0.20	0.20	0.20	0.20	0.20	0.01
74	MK039590	0.20	0.20	0.20	0.20	0.20	0.20	0.20	0.20	0.20	0.20	0.20	0.20	0.20	0.20	0.20	0.20	0.20	0.20	0.20	0.01
75	KT258661	0.20	0.20	0.20	0.20	0.20	0.20	0.20	0.20	0.20	0.20	0.20	0.20	0.20	0.20	0.20	0.20	0.20	0.20	0.20	0.01
76	KT258660	0.15	0.15	0.15	0.15	0.15	0.15	0.15	0.15	0.15	0.15	0.15	0.15	0.15	0.15	0.15	0.15	0.15	0.15	0.15	0.13
77	KT258659	0.15	0.15	0.15	0.15	0.15	0.15	0.15	0.15	0.15	0.15	0.15	0.15	0.15	0.15	0.15	0.15	0.15	0.15	0.15	0.13
78	KT258658	0.15	0.15	0.15	0.15	0.15	0.15	0.15	0.15	0.15	0.15	0.15	0.15	0.15	0.15	0.15	0.15	0.15	0.15	0.15	0.13
79	KT258657	0.15	0.15	0.15	0.15	0.15	0.15	0.15	0.15	0.15	0.15	0.15	0.15	0.15	0.15	0.15	0.15	0.15	0.15	0.15	0.13
80	KT258656	0.15	0.15	0.15	0.15	0.15	0.15	0.15	0.15	0.15	0.15	0.15	0.15	0.15	0.15	0.15	0.15	0.15	0.15	0.15	0.13

(continued on next table)

Table S1. (continued)

		61	62	63	64	65	66	67	68	69	70	71	72	73	74	75	76	77	78	79	80
61	OP899402																				
62	OP899401																				
63	OP899400	0.01																			
64	OP899399	0.01	0.01																		
65	OP899398	0.01	0.01	0.00																	
66	OP899397	0.01	0.01	0.01	0.00																
67	OP899395	0.01	0.00	0.01	0.01	0.01															
68	MT021781	0.01	0.01	0.00	0.00	0.00	0.01														
69	MK039635	0.02	0.01	0.01	0.01	0.01	0.01	0.01													
70	MK039594	0.01	0.01	0.01	0.01	0.01	0.01	0.01	0.01												
71	MK039593	0.01	0.01	0.01	0.01	0.01	0.01	0.01	0.01	0.00											
72	MK039592	0.01	0.01	0.01	0.01	0.01	0.01	0.01	0.01	0.00	0.00										
73	MK039591	0.02	0.01	0.01	0.01	0.01	0.01	0.01	0.00	0.01	0.01	0.01									
74	MK039590	0.01	0.01	0.01	0.01	0.01	0.01	0.01	0.01	0.00	0.00	0.00	0.01								
75	KT258661	0.01	0.01	0.01	0.01	0.01	0.01	0.01	0.01	0.00	0.00	0.00	0.01	0.00							
76	KT258660	0.13	0.12	0.12	0.12	0.12	0.12	0.12	0.12	0.12	0.12	0.12	0.12	0.12	0.12						
77	KT258659	0.13	0.12	0.13	0.12	0.12	0.12	0.12	0.12	0.12	0.12	0.12	0.12	0.12	0.12	0.00					
78	KT258658	0.13	0.12	0.12	0.12	0.12	0.12	0.12	0.12	0.12	0.12	0.12	0.12	0.12	0.12	0.00	0.01				
79	KT258657	0.13	0.12	0.12	0.12	0.12	0.12	0.12	0.12	0.12	0.12	0.12	0.12	0.12	0.12	0.00	0.00	0.00			
80	KT258656	0.12	0.11	0.12	0.12	0.11	0.11	0.12	0.11	0.12	0.12	0.12	0.11	0.12	0.12	0.02	0.02	0.02	0.02		

(continued on next table)

Table S1. (continued)

		1	2	3	4	5	6	7	8	9	10	11	12	13	14	15	16	17	18	19	20
81	KT258655	0.18	0.18	0.16	0.16	0.16	0.15	0.15	0.15	0.15	0.15	0.15	0.15	0.15	0.15	0.15	0.22	0.22	0.01	0.17	0.17
82	KT258654	0.18	0.19	0.17	0.17	0.17	0.15	0.15	0.15	0.15	0.15	0.15	0.15	0.16	0.15	0.15	0.22	0.21	0.01	0.16	0.16
83	KT258653	0.18	0.19	0.17	0.17	0.17	0.15	0.15	0.15	0.15	0.15	0.15	0.15	0.16	0.15	0.15	0.22	0.22	0.01	0.17	0.17
84	KT258652	0.18	0.19	0.17	0.17	0.17	0.15	0.15	0.15	0.15	0.15	0.15	0.15	0.16	0.15	0.15	0.22	0.22	0.01	0.16	0.16
85	KT258651	0.18	0.18	0.17	0.17	0.17	0.15	0.15	0.15	0.15	0.15	0.15	0.15	0.16	0.15	0.15	0.22	0.21	0.01	0.17	0.17
86	KT258650	0.18	0.18	0.17	0.17	0.17	0.15	0.15	0.15	0.15	0.15	0.15	0.15	0.16	0.15	0.15	0.22	0.21	0.01	0.17	0.17
87	KT258649	0.19	0.18	0.16	0.16	0.16	0.14	0.14	0.16	0.16	0.16	0.16	0.16	0.16	0.16	0.16	0.20	0.19	0.10	0.13	0.13
88	KT258648	0.19	0.18	0.16	0.16	0.16	0.14	0.14	0.16	0.16	0.16	0.16	0.16	0.16	0.16	0.16	0.20	0.19	0.10	0.13	0.13
89	KT258647	0.18	0.18	0.16	0.16	0.16	0.15	0.15	0.15	0.15	0.15	0.15	0.15	0.15	0.15	0.15	0.22	0.22	0.01	0.17	0.17
90	KT258646	0.18	0.19	0.17	0.17	0.17	0.15	0.15	0.15	0.15	0.15	0.15	0.15	0.16	0.15	0.15	0.22	0.22	0.01	0.17	0.17
91	KT258645	0.18	0.18	0.16	0.16	0.16	0.15	0.15	0.15	0.15	0.15	0.15	0.15	0.15	0.15	0.15	0.22	0.22	0.01	0.17	0.17
92	KT258644	0.18	0.18	0.16	0.16	0.16	0.15	0.15	0.15	0.15	0.15	0.15	0.15	0.15	0.15	0.15	0.22	0.22	0.01	0.17	0.17
93	KT258643	0.18	0.18	0.16	0.16	0.16	0.15	0.15	0.15	0.15	0.15	0.15	0.15	0.15	0.15	0.15	0.22	0.22	0.01	0.17	0.17
94	KT258642	0.18	0.18	0.16	0.16	0.16	0.15	0.15	0.15	0.15	0.15	0.15	0.15	0.15	0.15	0.15	0.22	0.22	0.01	0.17	0.17
95	KT258641	0.18	0.19	0.17	0.17	0.17	0.16	0.16	0.16	0.16	0.16	0.16	0.16	0.16	0.16	0.16	0.23	0.22	0.02	0.17	0.17
96	KT258640	0.18	0.18	0.16	0.16	0.16	0.15	0.15	0.15	0.15	0.15	0.15	0.15	0.15	0.15	0.15	0.22	0.22	0.01	0.17	0.17
97	KT258639	0.19	0.19	0.17	0.17	0.17	0.16	0.16	0.16	0.16	0.16	0.16	0.16	0.16	0.16	0.16	0.22	0.22	0.02	0.17	0.17
98	KT258638	0.18	0.18	0.16	0.16	0.16	0.15	0.15	0.15	0.15	0.15	0.15	0.15	0.15	0.15	0.15	0.22	0.22	0.01	0.17	0.17
99	KT258637	0.18	0.18	0.16	0.16	0.16	0.15	0.15	0.15	0.15	0.15	0.15	0.15	0.15	0.15	0.15	0.22	0.22	0.01	0.17	0.17
100	KT258636	0.18	0.18	0.16	0.16	0.16	0.15	0.15	0.15	0.15	0.15	0.15	0.15	0.15	0.15	0.15	0.22	0.22	0.01	0.17	0.17

(continued on next table)

Table S1. (continued)

		21	22	23	24	25	26	27	28	29	30	31	32	33	34	35	36	37	38	39	40
81	KT258655	0.12	0.15	0.15	0.15	0.15	0.15	0.15	0.15	0.15	0.15	0.15	0.15	0.15	0.15	0.15	0.15	0.15	0.15	0.15	0.15
82	KT258654	0.12	0.15	0.15	0.15	0.15	0.15	0.15	0.15	0.15	0.15	0.15	0.15	0.15	0.15	0.15	0.15	0.15	0.15	0.15	0.15
83	KT258653	0.12	0.15	0.15	0.15	0.15	0.15	0.15	0.15	0.15	0.15	0.15	0.15	0.15	0.15	0.15	0.15	0.15	0.15	0.15	0.15
84	KT258652	0.12	0.15	0.15	0.15	0.15	0.15	0.15	0.15	0.15	0.15	0.15	0.15	0.15	0.15	0.15	0.15	0.15	0.15	0.15	0.15
85	KT258651	0.12	0.15	0.15	0.15	0.15	0.15	0.15	0.15	0.15	0.15	0.15	0.15	0.15	0.15	0.15	0.15	0.15	0.15	0.15	0.15
86	KT258650	0.12	0.15	0.15	0.15	0.15	0.15	0.15	0.15	0.15	0.15	0.15	0.15	0.15	0.15	0.15	0.15	0.15	0.15	0.15	0.15
87	KT258649	0.13	0.16	0.16	0.16	0.16	0.16	0.16	0.16	0.16	0.16	0.16	0.16	0.16	0.16	0.16	0.16	0.16	0.16	0.16	0.16
88	KT258648	0.13	0.16	0.16	0.16	0.16	0.16	0.16	0.16	0.16	0.16	0.16	0.16	0.16	0.16	0.16	0.16	0.16	0.16	0.16	0.16
89	KT258647	0.12	0.15	0.15	0.15	0.15	0.15	0.15	0.15	0.15	0.15	0.15	0.15	0.15	0.15	0.15	0.15	0.15	0.15	0.15	0.15
90	KT258646	0.12	0.15	0.15	0.15	0.15	0.15	0.15	0.15	0.15	0.15	0.15	0.15	0.15	0.15	0.15	0.15	0.15	0.15	0.15	0.15
91	KT258645	0.12	0.15	0.15	0.15	0.15	0.15	0.15	0.15	0.15	0.15	0.15	0.15	0.15	0.15	0.15	0.15	0.15	0.15	0.15	0.15
92	KT258644	0.12	0.15	0.15	0.15	0.15	0.15	0.15	0.15	0.15	0.15	0.15	0.15	0.15	0.15	0.15	0.15	0.15	0.15	0.15	0.15
93	KT258643	0.12	0.15	0.15	0.15	0.15	0.15	0.15	0.15	0.15	0.15	0.15	0.15	0.15	0.15	0.15	0.15	0.15	0.15	0.15	0.15
94	KT258642	0.12	0.15	0.15	0.15	0.15	0.15	0.15	0.15	0.15	0.15	0.15	0.15	0.15	0.15	0.15	0.15	0.15	0.15	0.15	0.15
95	KT258641	0.12	0.16	0.16	0.16	0.16	0.16	0.16	0.16	0.16	0.16	0.16	0.16	0.16	0.16	0.16	0.16	0.16	0.15	0.16	0.16
96	KT258640	0.12	0.15	0.15	0.15	0.15	0.15	0.15	0.15	0.15	0.15	0.15	0.15	0.15	0.15	0.15	0.15	0.15	0.15	0.15	0.15
97	KT258639	0.11	0.16	0.16	0.16	0.16	0.16	0.16	0.16	0.16	0.16	0.16	0.16	0.16	0.16	0.16	0.16	0.16	0.15	0.16	0.16
98	KT258638	0.12	0.15	0.15	0.15	0.15	0.15	0.15	0.15	0.15	0.15	0.15	0.15	0.15	0.15	0.15	0.15	0.15	0.15	0.15	0.15
99	KT258637	0.12	0.15	0.15	0.15	0.15	0.15	0.15	0.15	0.15	0.15	0.15	0.15	0.15	0.15	0.15	0.15	0.15	0.15	0.15	0.15
100	KT258636	0.12	0.15	0.15	0.15	0.15	0.15	0.15	0.15	0.15	0.15	0.15	0.15	0.15	0.15	0.15	0.15	0.15	0.15	0.15	0.15

(continued on next table)

Table S1. (continued)

		41	42	43	44	45	46	47	48	49	50	51	52	53	54	55	56	57	58	59	60
81	KT258655	0.15	0.15	0.15	0.15	0.15	0.15	0.15	0.15	0.15	0.15	0.15	0.15	0.15	0.15	0.15	0.15	0.15	0.15	0.15	0.13
82	KT258654	0.15	0.15	0.15	0.15	0.15	0.15	0.15	0.15	0.15	0.15	0.15	0.15	0.15	0.15	0.15	0.15	0.15	0.15	0.15	0.13
83	KT258653	0.15	0.15	0.15	0.15	0.15	0.15	0.15	0.15	0.15	0.15	0.15	0.15	0.15	0.15	0.15	0.15	0.15	0.15	0.15	0.13
84	KT258652	0.15	0.15	0.15	0.15	0.15	0.15	0.15	0.15	0.15	0.15	0.15	0.15	0.15	0.15	0.15	0.15	0.15	0.15	0.15	0.13
85	KT258651	0.15	0.15	0.15	0.15	0.15	0.15	0.15	0.15	0.15	0.15	0.15	0.15	0.15	0.15	0.15	0.15	0.15	0.15	0.15	0.13
86	KT258650	0.15	0.15	0.15	0.15	0.15	0.15	0.15	0.15	0.15	0.15	0.15	0.15	0.15	0.15	0.15	0.15	0.15	0.15	0.15	0.13
87	KT258649	0.16	0.16	0.16	0.16	0.16	0.16	0.16	0.16	0.16	0.16	0.16	0.16	0.16	0.16	0.16	0.16	0.16	0.16	0.16	0.13
88	KT258648	0.16	0.16	0.16	0.16	0.16	0.16	0.16	0.16	0.16	0.16	0.16	0.16	0.16	0.16	0.16	0.16	0.16	0.16	0.16	0.13
89	KT258647	0.15	0.15	0.15	0.15	0.15	0.15	0.15	0.15	0.15	0.15	0.15	0.15	0.15	0.15	0.15	0.15	0.15	0.15	0.15	0.13
90	KT258646	0.15	0.15	0.15	0.15	0.15	0.15	0.15	0.15	0.15	0.15	0.15	0.15	0.15	0.15	0.15	0.15	0.15	0.15	0.15	0.13
91	KT258645	0.15	0.15	0.15	0.15	0.15	0.15	0.15	0.15	0.15	0.15	0.15	0.15	0.15	0.15	0.15	0.15	0.15	0.15	0.15	0.13
92	KT258644	0.15	0.15	0.15	0.15	0.15	0.15	0.15	0.15	0.15	0.15	0.15	0.15	0.15	0.15	0.15	0.15	0.15	0.15	0.15	0.13
93	KT258643	0.15	0.15	0.15	0.15	0.15	0.15	0.15	0.15	0.15	0.15	0.15	0.15	0.15	0.15	0.15	0.15	0.15	0.15	0.15	0.13
94	KT258642	0.15	0.15	0.15	0.15	0.15	0.15	0.15	0.15	0.15	0.15	0.15	0.15	0.15	0.15	0.15	0.15	0.15	0.15	0.15	0.13
95	KT258641	0.16	0.16	0.16	0.16	0.16	0.16	0.16	0.16	0.16	0.16	0.16	0.16	0.16	0.16	0.16	0.16	0.16	0.16	0.16	0.13
96	KT258640	0.15	0.15	0.15	0.15	0.15	0.15	0.15	0.15	0.15	0.15	0.15	0.15	0.15	0.15	0.15	0.15	0.15	0.15	0.15	0.13
97	KT258639	0.16	0.16	0.16	0.16	0.16	0.16	0.16	0.16	0.16	0.16	0.16	0.16	0.16	0.16	0.16	0.16	0.16	0.16	0.16	0.12
98	KT258638	0.15	0.15	0.15	0.15	0.15	0.15	0.15	0.15	0.15	0.15	0.15	0.15	0.15	0.15	0.15	0.15	0.15	0.15	0.15	0.13
99	KT258637	0.15	0.15	0.15	0.15	0.15	0.15	0.15	0.15	0.15	0.15	0.15	0.15	0.15	0.15	0.15	0.15	0.15	0.15	0.15	0.13
100	KT258636	0.15	0.15	0.15	0.15	0.15	0.15	0.15	0.15	0.15	0.15	0.15	0.15	0.15	0.15	0.15	0.15	0.15	0.15	0.15	0.13

(continued on next table)

Table S1. (continued)

		61	62	63	64	65	66	67	68	69	70	71	72	73	74	75	76	77	78	79	80
81	KT258655	0.13	0.12	0.12	0.12	0.12	0.12	0.12	0.12	0.12	0.12	0.12	0.12	0.12	0.12	0.00	0.00	0.00	0.00	0.02	0.00
82	KT258654	0.12	0.12	0.12	0.12	0.11	0.12	0.12	0.12	0.12	0.12	0.12	0.12	0.12	0.12	0.00	0.01	0.01	0.00	0.02	0.00
83	KT258653	0.13	0.12	0.13	0.12	0.12	0.12	0.12	0.12	0.12	0.12	0.12	0.12	0.12	0.12	0.00	0.00	0.01	0.00	0.02	0.00
84	KT258652	0.12	0.12	0.12	0.12	0.11	0.12	0.12	0.12	0.12	0.12	0.12	0.12	0.12	0.12	0.00	0.01	0.01	0.00	0.02	0.00
85	KT258651	0.13	0.12	0.13	0.12	0.12	0.12	0.12	0.12	0.12	0.12	0.12	0.12	0.12	0.12	0.00	0.01	0.01	0.00	0.02	0.00
86	KT258650	0.13	0.12	0.13	0.12	0.12	0.12	0.12	0.12	0.12	0.12	0.12	0.12	0.12	0.12	0.00	0.01	0.01	0.00	0.02	0.00
87	KT258649	0.13	0.13	0.13	0.13	0.12	0.13	0.13	0.13	0.13	0.13	0.13	0.13	0.13	0.13	0.09	0.09	0.09	0.09	0.09	0.09
88	KT258648	0.13	0.13	0.13	0.13	0.12	0.13	0.13	0.13	0.13	0.13	0.13	0.13	0.13	0.13	0.09	0.09	0.09	0.09	0.09	0.09
89	KT258647	0.13	0.12	0.12	0.12	0.12	0.12	0.12	0.12	0.12	0.12	0.12	0.12	0.12	0.12	0.00	0.00	0.00	0.00	0.02	0.00
90	KT258646	0.13	0.12	0.13	0.12	0.12	0.12	0.12	0.12	0.12	0.12	0.12	0.12	0.12	0.12	0.00	0.00	0.01	0.00	0.02	0.00
91	KT258645	0.13	0.12	0.12	0.12	0.12	0.12	0.12	0.12	0.12	0.12	0.12	0.12	0.12	0.12	0.00	0.00	0.00	0.00	0.02	0.00
92	KT258644	0.13	0.12	0.12	0.12	0.12	0.12	0.12	0.12	0.12	0.12	0.12	0.12	0.12	0.12	0.00	0.00	0.00	0.00	0.02	0.00
93	KT258643	0.13	0.12	0.12	0.12	0.12	0.12	0.12	0.12	0.12	0.12	0.12	0.12	0.12	0.12	0.00	0.00	0.00	0.00	0.02	0.00
94	KT258642	0.13	0.12	0.12	0.12	0.12	0.12	0.12	0.12	0.12	0.12	0.12	0.12	0.12	0.12	0.00	0.00	0.00	0.00	0.02	0.00
95	KT258641	0.13	0.13	0.13	0.13	0.12	0.13	0.13	0.12	0.12	0.12	0.12	0.12	0.12	0.12	0.01	0.00	0.01	0.01	0.02	0.01
96	KT258640	0.13	0.12	0.12	0.12	0.12	0.12	0.12	0.12	0.12	0.12	0.12	0.12	0.12	0.12	0.00	0.00	0.00	0.00	0.02	0.00
97	KT258639	0.12	0.11	0.12	0.11	0.11	0.11	0.11	0.11	0.11	0.11	0.11	0.11	0.11	0.11	0.01	0.01	0.01	0.01	0.01	0.01
98	KT258638	0.13	0.12	0.12	0.12	0.12	0.12	0.12	0.12	0.12	0.12	0.12	0.12	0.12	0.12	0.00	0.00	0.00	0.00	0.02	0.00
99	KT258637	0.13	0.12	0.12	0.12	0.12	0.12	0.12	0.12	0.12	0.12	0.12	0.12	0.12	0.12	0.00	0.00	0.00	0.00	0.02	0.00
100	KT258636	0.13	0.12	0.12	0.12	0.12	0.12	0.12	0.12	0.12	0.12	0.12	0.12	0.12	0.12	0.00	0.00	0.00	0.00	0.02	0.00

(continued on next table)

Table S1. (continued)

		81	82	83	84	85	86	87	88	89	90	91	92	93	94	95	96	97	98	99	100
81	KT258655																				
82	KT258654	0.00																			
83	KT258653	0.00	0.01																		
84	KT258652	0.00	0.01	0.01																	
85	KT258651	0.00	0.01	0.01	0.01																
86	KT258650	0.00	0.01	0.01	0.01	0.00															
87	KT258649	0.09	0.09	0.09	0.09	0.09	0.09														
88	KT258648	0.09	0.09	0.09	0.09	0.09	0.09	0.00													
89	KT258647	0.00	0.00	0.00	0.00	0.00	0.00	0.09	0.09												
90	KT258646	0.00	0.01	0.00	0.01	0.01	0.01	0.09	0.09	0.00											
91	KT258645	0.00	0.00	0.00	0.00	0.00	0.00	0.09	0.09	0.00	0.00										
92	KT258644	0.00	0.00	0.00	0.00	0.00	0.00	0.09	0.09	0.00	0.00	0.00									
93	KT258643	0.00	0.00	0.00	0.00	0.00	0.00	0.09	0.09	0.00	0.00	0.00	0.00								
94	KT258642	0.00	0.00	0.00	0.00	0.00	0.00	0.09	0.09	0.00	0.00	0.00	0.00	0.00							
95	KT258641	0.01	0.01	0.00	0.01	0.01	0.01	0.10	0.10	0.01	0.00	0.01	0.01	0.01	0.01						
96	KT258640	0.00	0.00	0.00	0.00	0.00	0.00	0.09	0.09	0.00	0.00	0.00	0.00	0.00	0.00	0.01					
97	KT258639	0.01	0.00	0.01	0.01	0.01	0.01	0.09	0.09	0.01	0.01	0.01	0.01	0.01	0.01	0.01	0.01				
98	KT258638	0.00	0.00	0.00	0.00	0.00	0.00	0.09	0.09	0.00	0.00	0.00	0.00	0.00	0.00	0.01	0.00	0.01			
99	KT258637	0.00	0.00	0.00	0.00	0.00	0.00	0.09	0.09	0.00	0.00	0.00	0.00	0.00	0.00	0.01	0.00	0.01	0.00		
100	KT258636	0.00	0.00	0.00	0.00	0.00	0.00	0.09	0.09	0.00	0.00	0.00	0.00	0.00	0.00	0.01	0.00	0.01	0.00	0.00	

(continued on next table)

Table S1. (continued)

		1	2	3	4	5	6	7	8	9	10	11	12	13	14	15	16	17	18	19	20
101	KT258635	0.18	0.19	0.17	0.17	0.17	0.15	0.15	0.15	0.15	0.15	0.15	0.15	0.16	0.15	0.15	0.22	0.22	0.01	0.17	0.17
102	KP843860	0.19	0.20	0.20	0.20	0.20	0.18	0.18	0.18	0.18	0.18	0.18	0.18	0.19	0.18	0.18	0.20	0.21	0.16	0.00	0.00
103	KP192848	0.19	0.20	0.20	0.20	0.20	0.18	0.18	0.18	0.18	0.18	0.18	0.18	0.19	0.18	0.18	0.20	0.21	0.16	0.00	0.00
104	KU517192	0.17	0.16	0.15	0.15	0.15	0.13	0.13	0.17	0.17	0.17	0.17	0.17	0.17	0.17	0.17	0.19	0.18	0.13	0.13	0.13
105	MG988672	0.17	0.17	0.16	0.16	0.16	0.13	0.13	0.16	0.16	0.16	0.16	0.16	0.17	0.16	0.16	0.19	0.19	0.13	0.13	0.13
106	MK039634	0.17	0.17	0.16	0.16	0.16	0.13	0.13	0.16	0.16	0.16	0.16	0.16	0.17	0.16	0.16	0.19	0.19	0.13	0.13	0.13
107	MK039632	0.17	0.16	0.15	0.15	0.15	0.13	0.13	0.17	0.17	0.17	0.17	0.17	0.17	0.17	0.17	0.19	0.18	0.13	0.13	0.13
108	MK039599	0.17	0.16	0.15	0.15	0.15	0.13	0.13	0.17	0.17	0.17	0.17	0.17	0.17	0.17	0.17	0.19	0.18	0.13	0.13	0.13
109	MK039598	0.17	0.16	0.15	0.15	0.15	0.13	0.13	0.17	0.17	0.17	0.17	0.17	0.17	0.17	0.17	0.19	0.18	0.13	0.13	0.13
110	MK039596	0.17	0.16	0.15	0.15	0.15	0.13	0.13	0.17	0.17	0.17	0.17	0.17	0.17	0.17	0.17	0.19	0.18	0.13	0.13	0.13
111	MG988669	0.18	0.19	0.15	0.15	0.15	0.15	0.15	0.17	0.17	0.17	0.17	0.17	0.17	0.17	0.17	0.21	0.21	0.13	0.17	0.17
112	MK039613	0.20	0.19	0.20	0.20	0.20	0.19	0.19	0.20	0.20	0.20	0.20	0.20	0.21	0.20	0.20	0.12	0.12	0.20	0.20	0.20

(continued on next table)

Table S1. (continued)

		21	22	23	24	25	26	27	28	29	30	31	32	33	34	35	36	37	38	39	40
101	KT258635	0.12	0.15	0.15	0.15	0.15	0.15	0.15	0.15	0.15	0.15	0.15	0.15	0.15	0.15	0.15	0.15	0.15	0.15	0.15	0.15
102	KP843860	0.16	0.18	0.18	0.18	0.18	0.18	0.18	0.18	0.18	0.18	0.18	0.18	0.18	0.18	0.18	0.18	0.18	0.18	0.18	0.18
103	KP192848	0.16	0.18	0.18	0.18	0.18	0.18	0.18	0.18	0.18	0.18	0.18	0.18	0.18	0.18	0.18	0.18	0.18	0.18	0.18	0.18
104	KU517192	0.14	0.17	0.17	0.17	0.17	0.17	0.17	0.17	0.17	0.17	0.17	0.17	0.17	0.17	0.17	0.17	0.17	0.17	0.17	0.17
105	MG988672	0.15	0.16	0.16	0.16	0.16	0.16	0.16	0.16	0.16	0.16	0.16	0.16	0.16	0.16	0.16	0.16	0.16	0.16	0.16	0.16
106	MK039634	0.15	0.16	0.16	0.16	0.16	0.16	0.16	0.16	0.16	0.16	0.16	0.16	0.16	0.16	0.16	0.16	0.16	0.16	0.16	0.16
107	MK039632	0.14	0.17	0.17	0.17	0.17	0.17	0.17	0.17	0.17	0.17	0.17	0.17	0.17	0.17	0.17	0.17	0.17	0.17	0.17	0.17
108	MK039599	0.14	0.17	0.17	0.17	0.17	0.17	0.17	0.17	0.17	0.17	0.17	0.17	0.17	0.17	0.17	0.17	0.17	0.17	0.17	0.17
109	MK039598	0.14	0.17	0.17	0.17	0.17	0.17	0.17	0.17	0.17	0.17	0.17	0.17	0.17	0.17	0.17	0.17	0.17	0.17	0.17	0.17
110	MK039596	0.14	0.17	0.17	0.17	0.17	0.17	0.17	0.17	0.17	0.17	0.17	0.17	0.17	0.17	0.17	0.17	0.17	0.17	0.17	0.17
111	MG988669	0.17	0.17	0.17	0.17	0.17	0.17	0.17	0.17	0.17	0.17	0.17	0.17	0.17	0.17	0.17	0.17	0.17	0.17	0.17	0.17
112	MK039613	0.19	0.20	0.20	0.20	0.20	0.20	0.20	0.20	0.20	0.20	0.20	0.20	0.20	0.20	0.20	0.20	0.20	0.21	0.20	0.20

(continued on next table)

Table S1. (continued)

		41	42	43	44	45	46	47	48	49	50	51	52	53	54	55	56	57	58	59	60
101	KT258635	0.15	0.15	0.15	0.15	0.15	0.15	0.15	0.15	0.15	0.15	0.15	0.15	0.15	0.15	0.15	0.15	0.15	0.15	0.15	0.13
102	KP843860	0.18	0.18	0.18	0.18	0.18	0.18	0.18	0.18	0.18	0.18	0.18	0.18	0.18	0.18	0.18	0.18	0.18	0.18	0.18	0.17
103	KP192848	0.18	0.18	0.18	0.18	0.18	0.18	0.18	0.18	0.18	0.18	0.18	0.18	0.18	0.18	0.18	0.18	0.18	0.18	0.18	0.17
104	KU517192	0.17	0.17	0.17	0.17	0.17	0.17	0.17	0.17	0.17	0.17	0.17	0.17	0.17	0.17	0.17	0.17	0.17	0.17	0.17	0.15
105	MG988672	0.16	0.16	0.16	0.16	0.16	0.16	0.16	0.16	0.16	0.16	0.16	0.16	0.16	0.16	0.16	0.16	0.16	0.16	0.16	0.15
106	MK039634	0.16	0.16	0.16	0.16	0.16	0.16	0.16	0.16	0.16	0.16	0.16	0.16	0.16	0.16	0.16	0.16	0.16	0.16	0.16	0.15
107	MK039632	0.17	0.17	0.17	0.17	0.17	0.17	0.17	0.17	0.17	0.17	0.17	0.17	0.17	0.17	0.17	0.17	0.17	0.17	0.17	0.15
108	MK039599	0.17	0.17	0.17	0.17	0.17	0.17	0.17	0.17	0.17	0.17	0.17	0.17	0.17	0.17	0.17	0.17	0.17	0.17	0.17	0.15
109	MK039598	0.17	0.17	0.17	0.17	0.17	0.17	0.17	0.17	0.17	0.17	0.17	0.17	0.17	0.17	0.17	0.17	0.17	0.17	0.17	0.15
110	MK039596	0.17	0.17	0.17	0.17	0.17	0.17	0.17	0.17	0.17	0.17	0.17	0.17	0.17	0.17	0.17	0.17	0.17	0.17	0.17	0.15
111	MG988669	0.17	0.17	0.17	0.17	0.17	0.17	0.17	0.17	0.17	0.17	0.17	0.17	0.17	0.17	0.17	0.17	0.17	0.17	0.17	0.18
112	MK039613	0.20	0.20	0.20	0.20	0.20	0.20	0.20	0.20	0.20	0.20	0.20	0.20	0.20	0.20	0.20	0.20	0.20	0.20	0.20	0.20

(continued on next table)

Table S1. (continued)

		61	62	63	64	65	66	67	68	69	70	71	72	73	74	75	76	77	78	79	80
101	KT258635	0.13	0.12	0.13	0.12	0.12	0.12	0.12	0.12	0.12	0.12	0.12	0.12	0.12	0.12	0.00	0.00	0.01	0.00	0.02	0.00
102	KP843860	0.17	0.15	0.16	0.16	0.16	0.15	0.16	0.16	0.17	0.17	0.17	0.16	0.17	0.17	0.17	0.17	0.16	0.17	0.15	0.17
103	KP192848	0.17	0.15	0.16	0.16	0.16	0.15	0.16	0.16	0.17	0.17	0.17	0.16	0.17	0.17	0.17	0.17	0.16	0.17	0.15	0.17
104	KU517192	0.15	0.14	0.15	0.14	0.14	0.14	0.14	0.14	0.14	0.14	0.14	0.14	0.14	0.14	0.13	0.14	0.14	0.13	0.13	0.13
105	MG988672	0.15	0.15	0.15	0.15	0.15	0.15	0.15	0.15	0.15	0.15	0.15	0.15	0.15	0.15	0.13	0.13	0.13	0.13	0.13	0.13
106	MK039634	0.15	0.15	0.15	0.15	0.15	0.15	0.15	0.15	0.15	0.15	0.15	0.15	0.15	0.15	0.13	0.13	0.13	0.13	0.13	0.13
107	MK039632	0.15	0.14	0.15	0.14	0.14	0.14	0.14	0.14	0.14	0.14	0.14	0.14	0.14	0.14	0.13	0.14	0.14	0.13	0.13	0.13
108	MK039599	0.15	0.14	0.15	0.14	0.14	0.14	0.14	0.14	0.14	0.14	0.14	0.14	0.14	0.14	0.13	0.14	0.14	0.13	0.13	0.13
109	MK039598	0.15	0.14	0.15	0.14	0.14	0.14	0.14	0.14	0.14	0.14	0.14	0.14	0.14	0.14	0.13	0.14	0.14	0.13	0.13	0.13
110	MK039596	0.15	0.14	0.15	0.14	0.14	0.14	0.14	0.14	0.14	0.14	0.14	0.14	0.14	0.14	0.13	0.14	0.14	0.13	0.13	0.13
111	MG988669	0.17	0.17	0.17	0.17	0.17	0.17	0.17	0.17	0.17	0.17	0.17	0.17	0.17	0.17	0.13	0.14	0.14	0.13	0.14	0.13
112	MK039613	0.19	0.19	0.19	0.19	0.19	0.19	0.19	0.19	0.19	0.19	0.19	0.19	0.19	0.19	0.21	0.21	0.21	0.21	0.21	0.21

(continued on next table)

Table S1. (continued)

		81	82	83	84	85	86	87	88	89	90	91	92	93	94	95	96	97	98	99	100
101	KT258635	0.00	0.01	0.00	0.01	0.01	0.01	0.09	0.09	0.00	0.00	0.00	0.00	0.00	0.00	0.00	0.00	0.01	0.00	0.00	0.00
102	KP843860	0.17	0.16	0.17	0.16	0.17	0.17	0.13	0.13	0.17	0.17	0.17	0.17	0.17	0.17	0.17	0.17	0.17	0.17	0.17	0.17
103	KP192848	0.17	0.16	0.17	0.16	0.17	0.17	0.13	0.13	0.17	0.17	0.17	0.17	0.17	0.17	0.17	0.17	0.17	0.17	0.17	0.17
104	KU517192	0.13	0.14	0.14	0.14	0.13	0.13	0.04	0.04	0.13	0.14	0.13	0.13	0.13	0.13	0.14	0.13	0.13	0.13	0.13	0.13
105	MG988672	0.13	0.13	0.13	0.13	0.13	0.13	0.04	0.04	0.13	0.13	0.13	0.13	0.13	0.13	0.14	0.13	0.13	0.13	0.13	0.13
106	MK039634	0.13	0.13	0.13	0.13	0.13	0.13	0.04	0.04	0.13	0.13	0.13	0.13	0.13	0.13	0.14	0.13	0.13	0.13	0.13	0.13
107	MK039632	0.13	0.14	0.14	0.14	0.13	0.13	0.04	0.04	0.13	0.14	0.13	0.13	0.13	0.13	0.14	0.13	0.13	0.13	0.13	0.13
108	MK039599	0.13	0.14	0.14	0.14	0.13	0.13	0.04	0.04	0.13	0.14	0.13	0.13	0.13	0.13	0.14	0.13	0.13	0.13	0.13	0.13
109	MK039598	0.13	0.14	0.14	0.14	0.13	0.13	0.04	0.04	0.13	0.14	0.13	0.13	0.13	0.13	0.14	0.13	0.13	0.13	0.13	0.13
110	MK039596	0.13	0.14	0.14	0.14	0.13	0.13	0.04	0.04	0.13	0.14	0.13	0.13	0.13	0.13	0.14	0.13	0.13	0.13	0.13	0.13
111	MG988669	0.13	0.13	0.14	0.14	0.13	0.13	0.13	0.13	0.13	0.14	0.13	0.13	0.13	0.13	0.14	0.13	0.13	0.13	0.13	0.13
112	MK039613	0.21	0.21	0.21	0.21	0.21	0.21	0.21	0.21	0.21	0.21	0.21	0.21	0.21	0.21	0.22	0.21	0.21	0.21	0.21	0.21

(continued on next table)

Table S1. (continued)

		101	102	103	104	105	106	107	108	109	110	111	112
101	KT258635												
102	KP843860	0.17											
103	KP192848	0.17	0.00										
104	KU517192	0.14	0.13	0.13									
105	MG988672	0.13	0.13	0.13	0.00								
106	MK039634	0.13	0.13	0.13	0.00	0.00							
107	MK039632	0.14	0.13	0.13	0.00	0.00	0.00						
108	MK039599	0.14	0.13	0.13	0.00	0.00	0.00	0.00					
109	MK039598	0.14	0.13	0.13	0.00	0.00	0.00	0.00	0.00				
110	MK039596	0.14	0.13	0.13	0.00	0.00	0.00	0.00	0.00	0.00			
111	MG988669	0.14	0.17	0.17	0.12	0.12	0.12	0.12	0.12	0.12	0.12		
112	MK039613	0.21	0.20	0.20	0.20	0.20	0.20	0.20	0.20	0.20	0.20	0.20	
